# The combination of *Mycobacterium tuberculosis* fusion proteins LT33 and LT28 induced strong protective immunity in mice

**DOI:** 10.3389/fimmu.2024.1450124

**Published:** 2024-11-22

**Authors:** Pu He, Juan Wang, Daquan Tan, Lina Hu, Yanlin Ma, Youjun Mi, Fei Li, Tingting Zhang, Yunjie Du, Wenhua Zhang, Jixi Li, Lei Jiao, Bingdong Zhu

**Affiliations:** ^1^ State Key Laboratory for Animal Disease Control and Prevention and Lanzhou Center for Tuberculosis Research, Institute of Pathogen Biology, School of Basic Medical Sciences, Lanzhou University, Lanzhou, China; ^2^ Lanzhou Institute of Biological Products, Lanzhou, China; ^3^ Institute of Pathogenic Physiology, School of Basic Medical Sciences, Lanzhou University, Lanzhou, China; ^4^ School of Life Science, Lanzhou University, Lanzhou, China; ^5^ State Key Laboratory of Genetic Engineering, School of Life Sciences, Fudan University, Shanghai, China; ^6^ College of Veterinary Medicine, Lanzhou University, Lanzhou Veterinary Research Institute, Chinese Academy of Agricultural Sciences, Lanzhou, China

**Keywords:** *Mycobacterium tuberculosis*, subunit vaccine, antigens, secreted antigen, latency-associated antigen

## Abstract

Effective subunit vaccines for tuberculosis (TB) must target antigenic components at various stages of infection. In this study, we constructed fusion proteins using secreted antigens from *Mycobacterium tuberculosis* (*M. tuberculosis*), specifically ESAT6, CFP10, MPT64, and Rv2645 from the proliferation stage, along with latency-associated antigens Rv1738 and Rv1978. The resulting fusion proteins, designated LT33 (ESAT6-CFP10-Rv1738) and LT28 (MPT64_61-170_-Rv1978_8-60_-Rv2645_21-80_), were combined with an adjuvant containing dimethyldioctadecylammonium bromide (DDA), polyriboinosinic polyribocytidylic acid (PolyI:C), and cholesterol to construct subunit vaccines. We evaluated the subunit vaccine effect in C57BL/6 mice and revealed that LT33 and LT28 exhibited strong immunogenicity and induced protective efficacy against aerosol challenge with *M. tuberculosis* H37Rv. Notably, the combination of LT33 and LT28 led to a significant reduction of 0.77 log10 colony-forming units (CFU) of H37Rv in the lungs compared to the adjuvant control group, highlighting their potential as promising candidates for subunit vaccine against *M. tuberculosis* infection.

## 1 Introduction

Tuberculosis (TB) is an infectious disease mainly caused by *Mycobacterium tuberculosis*, which causes approximately 10 million cases each year around the world. Bacillus Calmette-Guerin (BCG), when administered to newborns, can prevent children against severe TB infection for approximately 10–15 years (1, 2). The efficacy of BCG may diminish over time, and the TB subunit vaccine is supposed to have the potential to enhance BCG-primed immunity. However, MVA85A failed to improve the protection of BCG against TB as a booster for BCG, possibly due to limited antigen and an imperfect boosting schedule (3). Currently, there are at least five protein subunit vaccines undergoing clinical trials: M72/AS01E (4), ID93/GLA-SE (5), H56/IC31 (6), AEC/BC02 (7), and GamTBvac (8). Among these, M72/AS01E, comprising antigens Rv1196 and Rv0125, showed 49.7% efficacy in preventing active TB in HIV-uninfected individuals with latent *M. tuberculosis* infection over a 3-year clinical trial period (9).

The selection of multistage antigens from replicating and dormant stages represents a promising strategy to improve the efficacy of subunit vaccine. Several innovative multistage subunit vaccines, such as H56 and ID93 (10), have been developed. Notably, H56/CAF01, comprising early antigens Ag85B, ESAT6, and latency-associated protein Rv2660c, confers superior protection compared to H4:IC31 (11). Our lab previously constructed several fusion proteins containing multistage antigens, such as Mtb10.4-HspX (MH) (12), ESAT6-Ag85B-MPT64_190–198_-Mtb8.4-HspX (LT69), and ESAT6-Ag85B-MPT64_190–198_-Mtb8.4-Rv2626c (LT70). Among them, LT69 and LT70 induced strong protective efficacy compared to the PBS control at 30 weeks post-vaccination, approaching the level of protection induced by BCG (13, 14).

In comparison to *M. tuberculosis*, certain BCG sub-strains exhibit deletions in 16 genomic regions known as regions of difference (RD) (15). RD1 is present in clinical *M. tuberculosis* isolates and absent in all BCG sub-strains (16). The early secreted proteins ESAT6 and CFP10 within RD1 can modulate host immune response (17). ESAT6 induced the highest interferon (IFN)-γ secretion in pulmonary TB patients’ peripheral blood among 1,250 identified proteins (18). ESAT6 also induced antigen-specific CD4 and CD8 T cells, enhancing protection against *M. tuberculosis* infection (19). Vaccines containing ESAT6 provided protection in both pre-exposure and post-exposure models (20). Antigens from RD2 and RD13 also demonstrated significant potential as candidates for TB vaccines (21, 22). MPT64 and Rv1978 from RD2 elicited *M. tuberculosis*-specific IFN-γ responses in active TB patients (23) and latent TB individuals (LTBIs) (21). As a diagnostic antigen for TB, Rv2645 from RD13 could also stimulate peripheral blood mononuclear cells (PBMCs) from TB patients producing IFN-γ (24). The recombinant BCG::Rv2645 generated superior protective immunity compared to BCG (22). In addition, Rv1738 exhibited high upregulation in dormant bacteria (25) and induced significant IFN-γ production in *M. tuberculosis* close contacts (26).

In this study, we developed two fusion proteins, LT33 and LT28. LT33 is composed of RD antigens ESAT6, CFP10, and the latency-associated protein Rv1738, while LT28 contains fragments of RD antigens MPT64, Rv1978, and Rv2645. These fusion proteins were combined with an adjuvant DPC [comprising dimethyldioctadecylammonium bromide (DDA), polyriboinosinic polyribocytidylic acid (PolyI:C), and cholesterol], and their immunogenicity and protective efficacy were assessed in C57BL/6 mice.

## 2 Materials and methods

### 2.1 Animals and ethics statement

C57BL/6 (6–8 weeks old) female mice were purchased from Gansu University of Chinese Medicine (Lanzhou, China) and Vital River company (Beijing, China), which were raised under specific pathogen-free conditions at Lanzhou University or the Lanzhou Institute of Biological Products (Lanzhou, China). The vaccinated mice were exposed to challenge with *M. tuberculosis* H37Rv strains in the ABSL-3 Laboratory by Gene optimal (Foshan, China).

All animal experiments were carried out under the guidelines of the Council on Animal Care and Use, with protocols approved by the Institutional Animal Care and Use Committee of Lanzhou University. Animals were monitored daily and received free access to water and food throughout the study. Mice were euthanized by cervical dislocation.

### 2.2 Bacterial strains

*M. tuberculosis* H37Ra (ATCC25177) and BCG (Danish strain) bacteria were donated by Fudan University and the Lanzhou Institute of Biological Products, respectively. They were cultured in Sauton’s medium and saved in our lab. *M. tuberculosis* H37Rv (NC_000962.3) strain was prepared in the ABSL-3 Laboratory by Gene optimal (Foshan, China).

### 2.3 Construction and predicting the structure of fusion proteins

**Figure 1:**
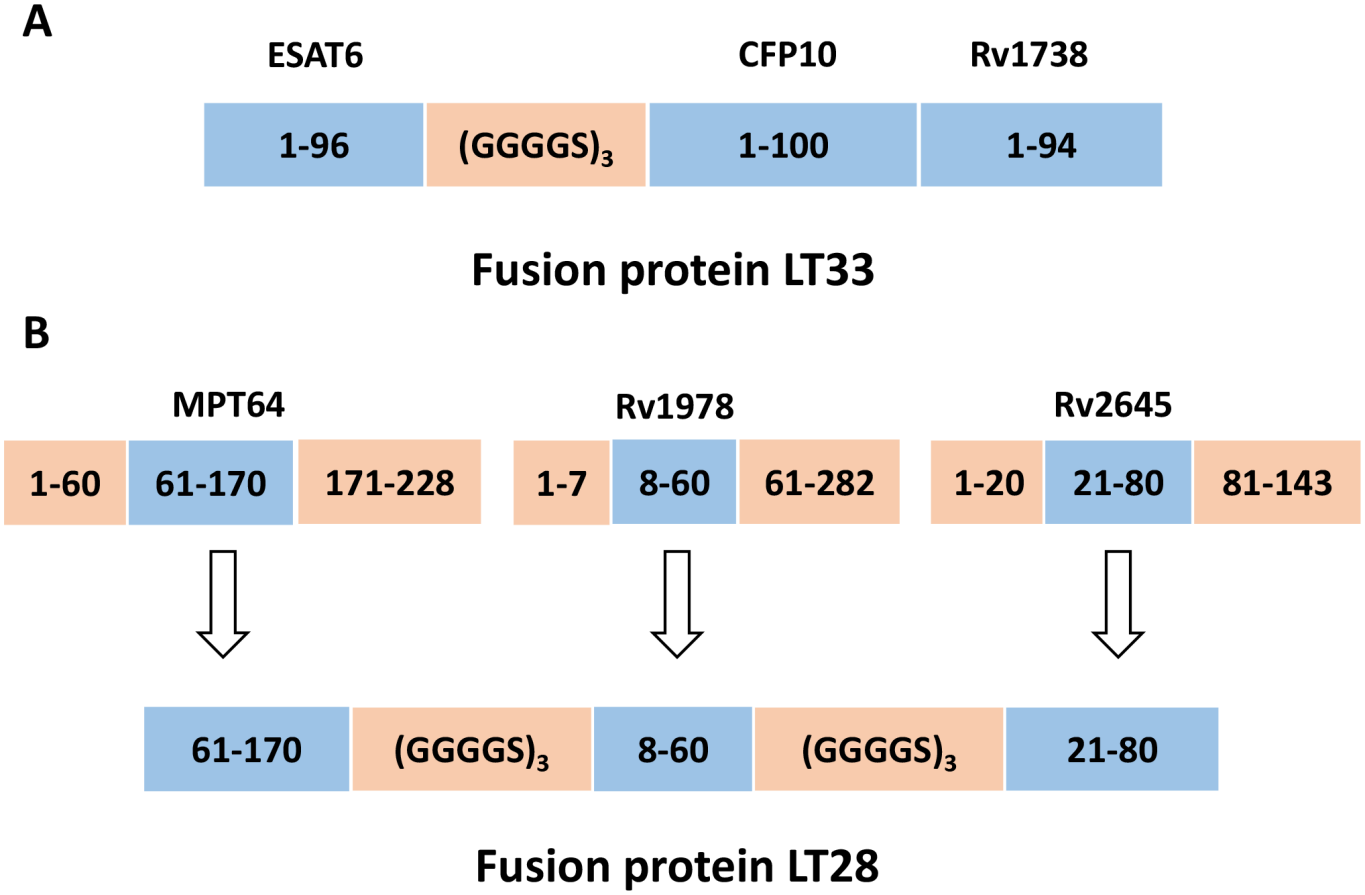
Construction of fusion protein LT33 and LT28. **(A)** The construction of LT33. ESAT6, CFP10, and Rv1738 were utilized in the creation of fusion protein LT33, in which ESAT6 and CFP10 were connected through a linker. **(B)** After analyzing the immunodominant epitopes and hydrophilic fragments of antigens, the MPT64(61–170 aa), Rv1978(8–60 aa), and Rv2645(21–80 aa) fragments were selected for the construction of fusion protein LT28.

Recombinant pET30a (+)-ESAT6-CFP10 were produced as previously described (27). ESAT6 and CFP10 were linked together using a flexible linker consisting of three repeats of GGGGS. The plasmid encoding ESAT6-CFP10-Rv1738 (designated LT33) was created by inserting the gene Rv1738 into the vector pET30a (+)-ESAT6-CFP10 (**Figure 1A**). The final plasmid constructed was transformed into the *Escherichia coli* strain BL21 for the production of the fusion protein LT33.

The dominant epitopes and hydrophilic fragments of MPT64, Rv1978, and Rv2645 were selected to construct the fusion protein LT28. The dominant epitopes for T and B cells were predicted using the Immune Epitope Database and Analysis Resource (IEDB, http://www.iedb.org/) (**Table 1**), while hydrophilic fragments were predicted using ProtScale (https://web.expasy.org/protscale/). The amino acid sequences were obtained from the National Center for Biotechnology Information (NCBI, https://www.ncbi.nlm.nih.gov/) database. The gene sequence was synthesized by BGI Genomics Co., Ltd. (Shenzhen, China) and the synthesized fragments were inserted into the unique restriction sites *Nde* I and *Hin*d III of the pET30a (+) vector to construct the plasmid pET30a (+)-MPT64_(61-170)_-Rv1978_(8-60)_-Rv2645_(21-80)_. The adjacent epitope fragments MPT64_(61-170)_, Rv1978_(8-60)_, and Rv2645_(21-80)_ were linked together using the flexible linker (GGGGS)_3_ (**Figure 1B**). The final plasmid was then transformed into the *E. coli* strain BL21 for the production of the fusion protein LT28.

**Table 1:** The immunodominant epitopes of LT28 predicted by the IEDB database.

Antigen	Type of peptides	Location (aa)	Peptide sequence
MPT64	CD4^+^ T-cell epitopes	74–87	RDKFLSAATSSTPR
		87–101	REAPYELNITSATYQ
	CD8^+^ T-cell epitopes	85–93	TPREAPYEL
		122–130	GTHPTTTYK
		130–138	KAFDWDQAY
	B-cell epitopes	60–69	YPDQKSLENY
		81–92	ATSSTPREAPYE
		137–153	AYRKPITYDTLWQADTD
Rv1978	CD4^+^ T-cell epitopes	24–37	LDRRFQTDALEYLD
		25–40	DRRFQTDALEYLDRDD
	CD8^+^ T-cell epitopes	28–36	FQTDALEYL
		14–23	MPRGGPDASW
Rv2645	CD4^+^ T-cell epitopes	67–80	RYFPAGDPVAADVW
	CD8^+^ T-cell epitopes	33–41	GPATPPPPW
		47–56	EPIWEQLTER
		56–65	RYGGVTICQW
	B-cell epitopes	68–74	YFPAGDP

The structures of LT33 and LT28 were predicted using AlphaFold2 and the structures of the individual antigenic peptides within the fusion proteins were analyzed using USCF chimera software.

### 2.4 Expression and purification of fusion proteins

The fusion protein MH vaccines were prepared as previously described (12). *E. coli* BL21 containing the expressing plasmids was incubated with 0.5 mM isopropyl β-D-thiogalactopyanoside (IPTG) for 4 h at 37°C. The cells were then harvested and sonicated in phosphate buffer (20 mM; pH 7.4). Samples were sedimented by centrifugation at 10,000*g* for 10 min at 4°C to separate the inclusion body proteins from the soluble proteins.

The overexpressed LT33 remained in the supernatant in a soluble form. Saturated ammonium sulfate was added to the protein sample to achieve 25% saturation, and the supernatant was discarded by centrifugation. The precipitate containing the target protein was then resuspended in phosphate buffer (20 mM; pH 7.4). LT33 was further purified using ion-exchange chromatography on a Q-Sepharose high-performance column with an AKTA Purifier 150 (GE Healthcare, Piscataway, NJ). LT33 was eluted from the resin with phosphate buffer (20 mM; sodium chloride, 1 M; pH 7.4).

The overexpressed LT28 aggregated into inclusion bodies. To extract the protein, the inclusion bodies were dissolved in a solubilization buffer consisting of Tris-HCl (50 mM) and 3 M urea at pH 8.5. Following this, a step-wise dialysis method was employed to reduce the urea levels and facilitate the refolding of the protein structure. LT28 was then further purified using gel filtration chromatography (GFC) on a Superdex 75 pre-grade column, and LT28 was eluted from the resin with Tris-HCl buffer (50 mM; pH 8.5).

The purified LT33 and LT28 were assessed using SDS-PAGE and confirmed by Western blot with antigen-specific antibodies. The molecular weight and purity of the protein were determined through SDS-PAGE, utilizing the Bio-Rad Mini-PROTEAN Tetra Electrophoresis System (Bio-Rad, CA, USA).

### 2.5 Vaccine immunization program

**Figure 2:**
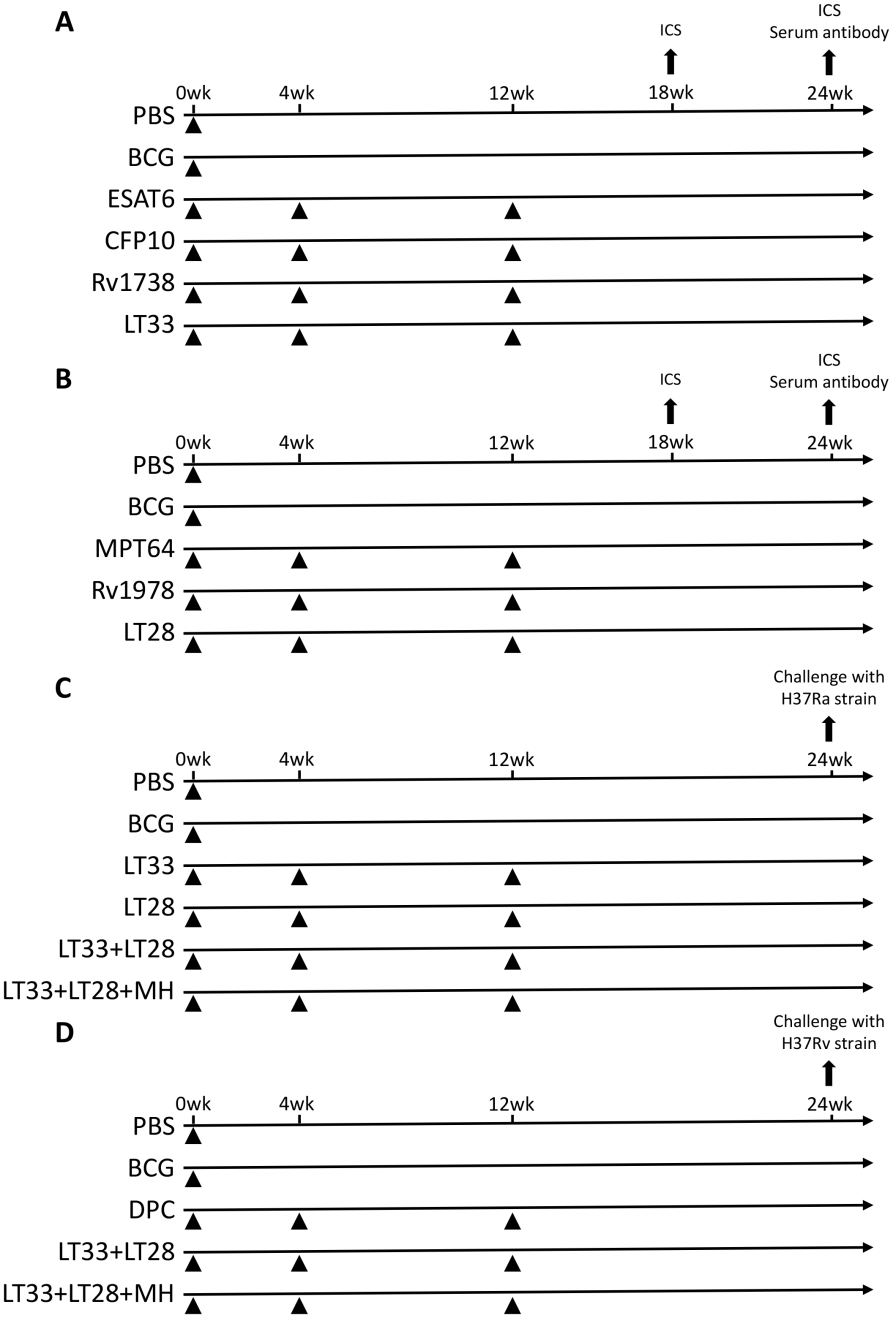
Vaccination program. **(A)** Immunization schedule of the LT33 vaccine for immunogenicity detection. **(B)** Immunization schedule of the LT28 vaccine for immunogenicity detection. **(C)** Immune program of an experimental vaccine against the H37Ra strain in mice. **(D)** Immune program of an experimental vaccine against the H37Rv strain in mice. ICS, intracellular cytokine staining.

The fusion protein LT33 or LT28 (10 μg/dose) was emulsified in an adjuvant consisting of DDA (250 μg/dose), PolyI:C (50 μg/dose), and cholesterol (75 μg/dose) to construct a subunit vaccine. To evaluate the immunogenicity of this vaccine, C57BL/6 mice were immunized subcutaneously with LT33 (10 μg/dose), ESAT6 (10 μg/dose), LT28 (10 μg/dose), MPT64 (10 μg/dose), and Rv1978 (10 μg/dose) at intervals of 0, 4, and 12 weeks. As a control, BCG [5 × 10^6^ colony-forming units (CFU) in 100 μL per mouse] was administered subcutaneously once at week 0 (**Figure 2**).

To assess the immune protective efficacy of LT33 and LT28 vaccines, C57BL/6 mice were immunized subcutaneously with various combinations: LT33 (10 μg/dose), LT28 (10 μg/dose), LT33 (5 μg/dose) + LT28 (5 μg/dose), and LT33 (3.3 μg/dose) + LT28 (3.3 μg/dose) + MH (3.3 μg/dose), administered three times at weeks 0, 4, and 12. Mice received BCG and PBS once at week 0 as positive and sham controls, respectively (**Figure 2**).

### 2.6 Detection of IFN-γ and IL-2 secretion in vaccine-immunized mice

Mice were euthanized, and mononuclear cells were isolated from the spleens of vaccine-immunized mice using Mouse 1 × Lymphocyte Separation Medium (Dakewe Biotech Company Limited, China). After treating the spleen of mice with lymphocyte separation solution, we obtained the mononuclear cells, predominantly composed of lymphocytes along with a few mononuclear macrophages and dendritic cells. The isolated mononuclear cells were cultured in RPMI-1640 medium supplemented with 10% fetal bovine serum (FBS) and 100 U/mL penicillin–streptomycin solution. Subsequently, the mononuclear cells were inoculated into 24-well plates at a density of 5 × 10^6^ cells per well and stimulated with single antigen at 37°C in a 5% CO_2_ environment. After 4 h of stimulation, the cells were incubated for an additional 7–8 h with BD GolgiPlug™ (containing brefeldin A) at 37°C in 5% CO_2_. The cells were then collected and stained with anti-CD4-FITC (RM4-5, eBioscience) and anti-CD8-PerCP-Cy5.5 (53-6.7, eBioscience). Lymphocytes were permeabilized using the BD Cytofix/Cytoperm kit according to the manufacturer’s instructions and stained with anti-IFN-γ-APC (XMG1.2, eBioscience) and anti-IL-2-PE (JES6-5H4, BD). Finally, lymphocytes from individual mice were analyzed using a NovoCyte flow cytometer (ACEA Biosciences). Flow cytometry gating strategy is shown in **Supplementary Figure S1**. The mean fluorescence intensity (MFI) of IFN-γ is shown in **Supplementary Figure S2**.

### 2.7 Detection of long-term T cell-mediated immune memory induced by vaccine immunization

The long-term T cell-mediated immune memory responses induced by LT33 and LT28 were analyzed as previously described (28). For the LT33 immunization, mice were injected subcutaneously with the antigen ESAT6 (10 μg/mouse) to promote the differentiation of T_CM_ like long-term memory T cells into T_EM_ or effector T cell (T_eff_) *in vivo*, 3 days before immune detection. After the designated period, the mice were euthanized, and splenocytes were isolated and stimulated *in vitro* with ESAT6 antigens (10 μg/mL) for 12 h. During this incubation, T_EM_ would develop into T_eff_ and secrete cytokine IFN-γ. Intracellular cytokine staining was performed and analyzed by flow cytometry to indirectly assess the functionality of the long-lived memory T cells.

Using the same methods, the long-term T cell-mediated immune memory induced by LT28 was assessed. Initially, the single-protein MPT64, Rv1978, and Rv3425 peptides were utilized to stimulate the LT28 immunized mice *in vivo*, 3 days prior to immune detection. Following this stimulation period, splenocytes were isolated from the mice and subjected to *in vitro* stimulation with specific antigens or peptides. Intracellular cytokine staining was then performed, and the resulting data were analyzed by flow cytometry to evaluate the immune memory response.

### 2.8 Analyzing antigen-specific antibodies in mouse sera by enzyme-linked immunosorbent assay

Blood samples were obtained from anesthetized mice and were subsequently incubated at ambient temperature for 1 h. Then, the samples were centrifuged at 3,000 rpm for 15 min to separate serum. Antigen-specific immunoglobulin IgG, IgG1, and IgG2c in sera were detected by indirect enzyme-linked immunosorbent assay (ELISA) 12 weeks after the last immunization. First, the plates were coated with 100 μL/well of ESAT6, CFP10, Rv1978, MPT64 protein, and Rv2645 peptides at 5 μg/mL in PBS overnight at 4°C. Second, the plates were blocked with 5% skimmed milk powder and then incubated with the double-diluted serum at 37°C for 1 h. Goat anti-mouse IgG (Solarbio, Beijing, China) and rabbit anti-mouse IgG1 and IgG2c (Rockland Immunochemicals Inc., Montgomery, PA, USA) were added with 100 µL/well. After washing, the 3,3′,5,5′-tetramethylbenzidine (TMB) substrate was added with 200 µL/well and incubated at room temperature for 5 min. The reaction was then stopped by diluted sulfuric acid (1 mol/L) at 50 μL/well. The color was quantified at 450 nm. The serum in the PBS group was used as the negative control. The antibody titer was evaluated as a reciprocal of each endpoint dilution.

### 2.9 *M. tuberculosis* challenge experiments

Twelve weeks after the final inoculation, the animals were challenged with the H37Ra strain (5×10^6^ CFU) through tail vein injection. The number of viable bacteria in the lungs and spleen was quantified in the next 3 weeks.

Meanwhile, 12 weeks after the last inoculation, six C57BL/6 mice each group were challenged with the H37Rv (10–1,000 CFU) strain through aerosol infection, and the survival of mice was monitored. Six weeks later, the number of viable bacteria in the lung and spleen was counted. The dilutions of the tissue samples were plated on Middlebrook 7H10 plates (BD) containing oleic acid/albumin/dextrose/catalase (OADC). The CFU were counted.

For the H37Rv challenge mice, some tissue samples were additionally processed and stained with hematoxylin and eosin (H&E) to evaluate pathological alterations. A pathologist conducted the evaluation in a blinded manner to ensure objectivity. Additionally, the software CaseViewer was utilized to quantify the proportion of lung tissue occupied by inflammatory cells, providing a detailed analysis of the inflammatory response within the samples.

### 2.10 Statistical analysis

Data were evaluated by the GraphPad Prism 8 Software (GraphPad Software, San Diego, CA, USA). Statistical significance was analyzed by one-way ANOVA followed by a Tukey *post-hoc* test. *p* < 0.05 was considered statistically significant.

## 3 Results

### 3.1 Preparation of mycobacterial fusion proteins

**Figure 3:**
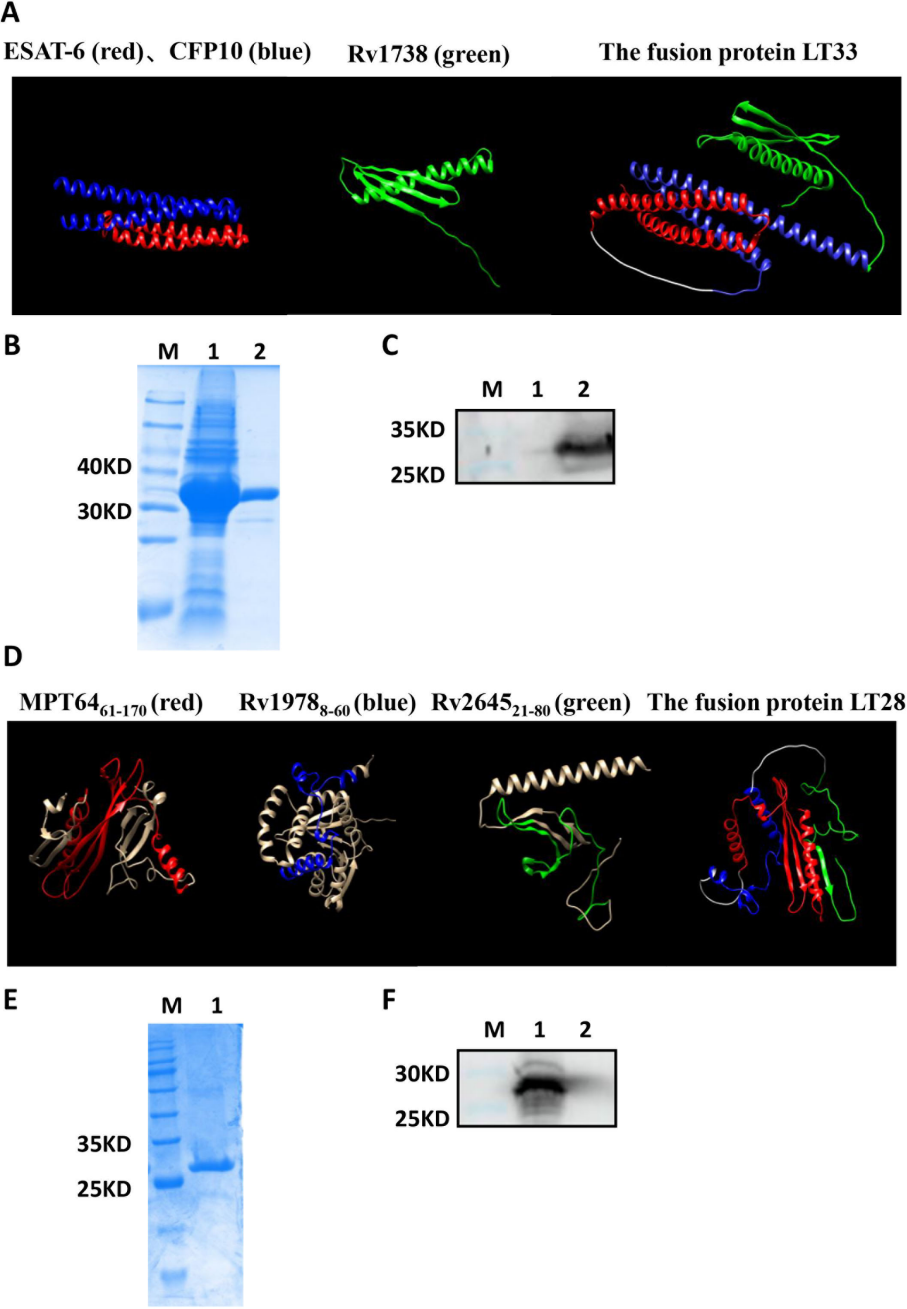
Construction of fusion protein LT33 and LT28. Expression, purification, and analysis of fusion protein LT33 and LT28. **(A)** The 3D structure of ESAT6 (red), CFP10 (blue), Rv1738 (green), and the fusion protein LT33. **(B)** Purification of LT33 verified with polyacrylamide gel electrophoresis. *E*. *coli* BL21 expressing LT33 lysate (lane 1), purification of LT33 (lane 2) **(C)** Purified LT33 was verified by immunoblot using anti-ESAT6 serum. Negative control (lane 1), mouse polyclonal anti-ESAT6 (lane 2), M, molecular weight. **(D)** The 3D structure of MPT64 (red), Rv1978 (blue), Rv2645 (green), and the fusion protein LT28. **(E)** Purification of LT28 verified with polyacrylamide gel electrophoresis. Purification of LT28 (lane 1). **(F)** Purified LT28 was verified by immunoblot with anti-MPT64 serum. Mouse polyclonal anti-MPT64 (lane 1), negative control (lane 2). M, molecular weight.

The fusion protein LT33 was composed of three *M. tuberculosis* antigens ESAT6, CFP10, and Rv1738, with a molecular weight of 33 kD. Predictions of the three-dimensional structure indicated that LT33 forms a stable complex structure, with the α-helices of ESAT6 and CFP10 and the structure of Rv1738 well-conserved within LT33 (**Figure 3A**). LT33 was produced in *E. coli* BL21 and purified using salting out and ion exchange chromatography (**Figure 3B**). The purified LT33 was confirmed by Western blot, which could be recognized by anti-ESAT6 antibody (**Figure 3C**).

Based on the epitope prediction results, three fragments MPT64(61–170), Rv1978(8–60), and Rv2645(21–80) were selected to construct the fusion protein LT28 (**Table 1**). The 3D structure prediction revealed that LT28 consists of α-helices and β-sheets linked by the flexible (GGGGS)_3_ linker (**Figure 3D**). LT28 was expressed in *E. coli* as inclusion bodies and purified by GFC successively (**Figure 3E**). The purified LT28, recognized by anti-MPT64 antibody, was validated *via* Western blot analysis (**Figure 3F**).

### 3.2 LT33 elicited robust cellular and humoral immune responses

**Figure 4:**
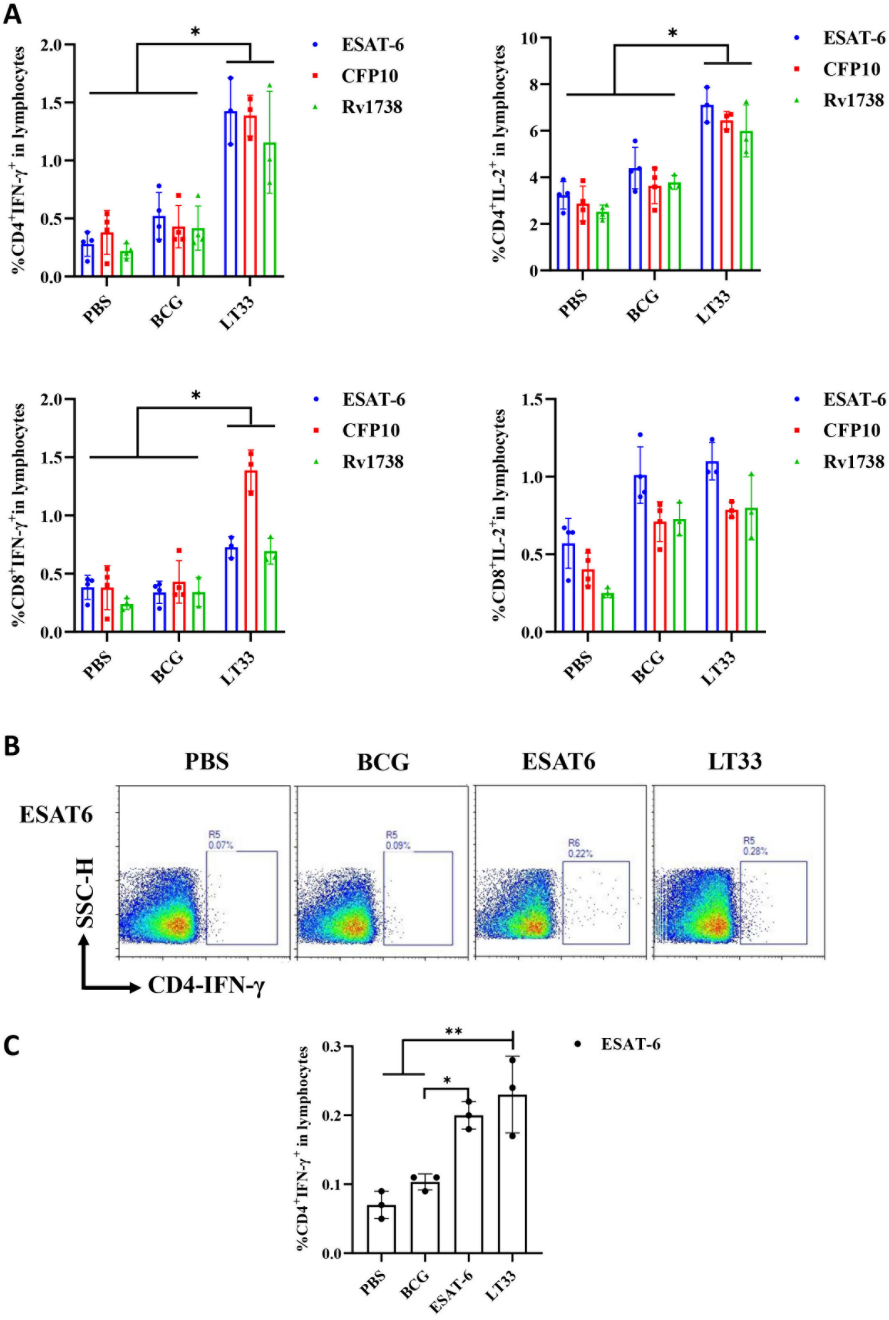
The immunogenicity of LT33 in mice. **(A)** At 6 weeks after the last immunization, the splenic lymphocytes were separated and stimulated with mixed antigens of ESAT6, CFP10, and Rv1738 *in vitro* for 12h. Flow cytometric analysis of IFN-γ and producing CD4 T cells and CD8 T cells. **(B)** At 12 weeks after the last immunization, the mice were stimulated with ESAT6 *in vivo* at 3 days before the mice were euthanized. Then, the splenic lymphocytes were separated and stimulated with ESAT6 *in vitro* for 12h. Intracellular cytokine staining was analyzed using flow cytometry. **(C)** Flow cytometric analysis of IFN-γ-producing CD4^+^ T cells by re-stimulation. The above panel is the representing figure. Results are presented as means ± SD, *n* = 3–4. *p < 0.05, **p < 0.01.

Six weeks after final vaccination, we assessed the functionality of LT33 vaccine-induced antigen-specific T cells. Compared to the control group, LT33 elicited a heightened presence of CD4^+^IFN-γ^+^, CD8^+^IFN-γ^+^, and CD4^+^IL-2^+^ T cells in the spleen, when stimulated with ESAT6, CFP10, and Rv1738 *in vitro* (**Figure 4A**, **Supplementary Figure S3**).

To observe the level of long-term memory T cells induced by the LT33 vaccine, the production of ESAT6-specific IFN-γ was assessed following a second antigen stimulation *in vivo* and *in vitro* at 3-day intervals by flow cytometry. The frequencies of IFN-γ-producing ESAT6-specific CD4 T cells in mice immunized with LT33 and ESAT6 were significantly higher than those in the BCG and PBS groups (*p* < 0.01; **Figures 4B, C**). There was no obvious difference between the former two groups, indicating that both LT33 and ESAT6 triggered antigen-specific cell-mediated immune responses. Furthermore, LT33-specific antibodies were measured at 12 weeks after the last injection, revealing measurable levels of antigen-specific IgG, IgG1, and IgG2c against ESAT6 (**Table 2**). These results demonstrate the high immunogenicity of LT33.

**Table 2:** The production of antigen-specific IgG, IgG1, and IgG2c induced by LT33.

	Groups	Antibody titers			
		IgG	IgG1	IgG2c	IgG2c/IgG1
	PBS	–	–	–	–
	BCG	–	–	–	–
Anti-ESAT-6	ESAT6	3.54 ± 0.43	4.22 ± 0.14	4.45 ± 0.03	1.055
	LT33	3.10 ± 0.05	4.15 ± 0.11	4.24 ± 0.09	1.022

At 12 weeks after the last immunization, the IgG, IgG1, and IgG2c against ESAT6 in serum were measured by ELISA. Data are expressed as means ± standard deviation (SD) (*n* = 3). Antibody titers are presented as the means of log10 antibody titers ± SD.

### 3.3 LT28 induced strong cellular and humoral immune responses

**Figure 5:**
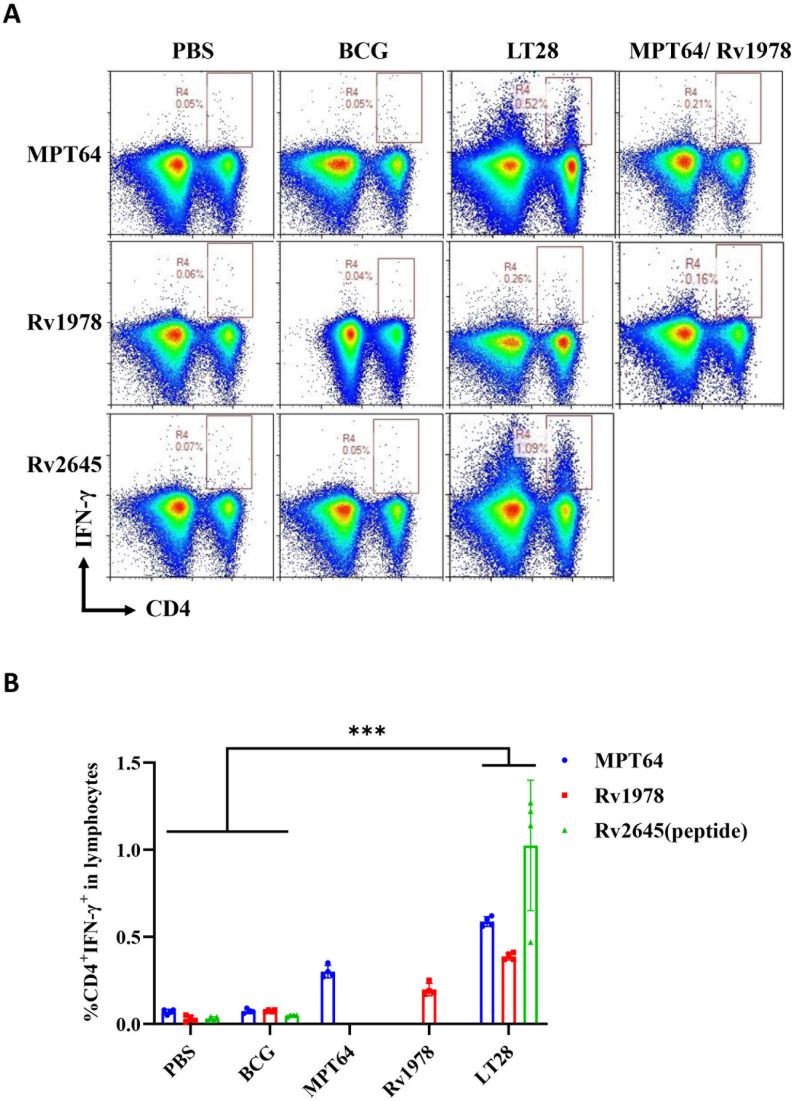
The immunogenicity of LT28 in mice. At 12 weeks after the last immunization, the mice were stimulated with a single antigen (MPT64, Rv1978, and Rv2645 peptide) *in vivo* at 3 days before the mice were euthanized. Then, the splenic lymphocytes were separated and stimulated with a single antigen (MPT64, Rv1978, and Rv2645) *in vitro* for 12h. Intracellular cytokine staining was analyzed using flow cytometry. **(A)** Flow cytometric analysis of IFN-γ-producing CD4^+^ T cells by re-stimulation. **(B)** Statistical analysis of the proportion of IFN-γ-producing CD4^+^ T cells. Results are presented as means ± SD, *n* = 4. ***p < 0.005.

The immunogenicity of LT28 was evaluated in C57BL/6 mice through vaccination with PBS, BCG, MPT64, Rv1978, and LT28, respectively. Twelve weeks after the final immunization, antigen-specific IFN-γ production was measured. Compared to PBS and BCG groups, the LT28 group induced higher numbers of antigen-specific IFN-γ-producing T cells after the second antigen stimulation. Similar responses were observed among LT28, MPT64, and Rv1978 groups following specific antigen stimulation (**Figure 5**). Mice immunized with LT28 also generated robust antigen-specific IL-2 production (**Supplementary Figure S4**). Additionally, the LT28 group generated high level of antibodies (IgG, IgG1, and IgG2c) against MPT64, Rv1978, and Rv2645 peptide pools (**Table 3**), suggesting that LT28 induces strong cellular and humoral immune responses.

**Table 3:** The production of antigen-specific IgG, IgG1, and IgG2c induced by LT28.

	Groups	Antibody titers			
		IgG	IgG1	IgG2c	IgG2c/IgG1
	PBS	–	–	–	–
	BCG	–	–	–	–
Anti-MPT64	MPT64	5.44 ± 0.51	4.85 ± 0.15	4.93 ± 0.05	1.016
	LT28	4.82 ± 0.14	4.53 ± 0.20	4.70 ± 0.29	1.037
Anti-Rv1978	Rv1978	4.88 ± 0.65	4.35 ± 0.15	3.92 ± 0.11	0.901
	LT28	4.55 ± 0.33	3.58 ± 0.08	4.33 ± 0.39	1.209
Anti-Rv2645	LT28	4.52 ± 0.27	4.30 ± 0.02	4.16 ± 0.37	0.967

At 12 weeks after the last immunization, the IgG, IgG1, and IgG2c against MPT64, Rv1978, and Rv2645 (peptide) in serum were measured by ELISA. Data are expressed as means ± standard deviation (SD) (*n* = 4). Antibody titers are presented as the means of log10 antibody titers ± SD.

### 3.4 The combination of LT33 and LT28 demonstrated effective immune protection

**Figure 6:**
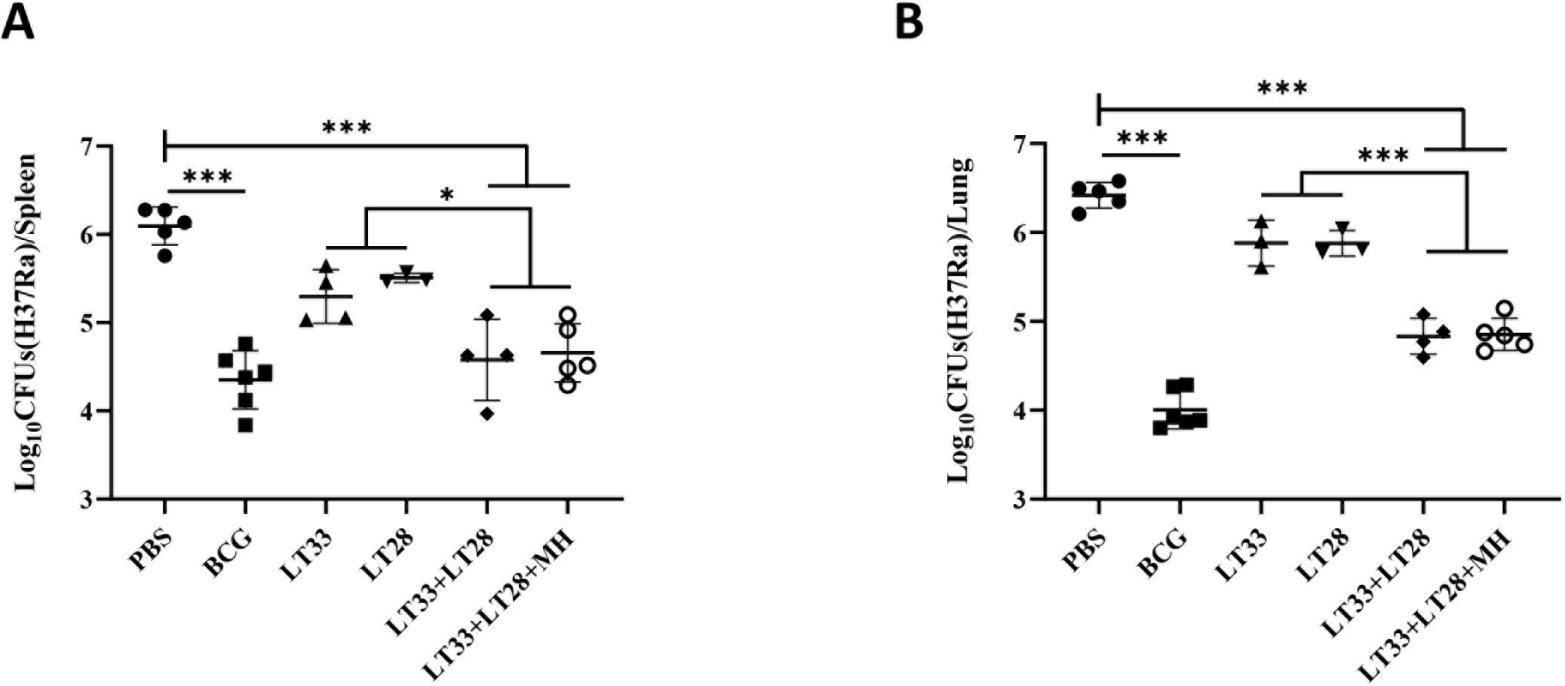
The protective efficacy of LT33 and LT28 challenged with the H37Ra strain. At 12 weeks after the last immunization, the C57BL/6 mice were intravenously challenged with the *M. tuberculosis* H37Ra strain. The protective efficacy was measured by detecting the bacteria load in lung tissues and spleen tissues. Mice were euthanized and the bacterial burden (H37Ra) was measured in the spleens **(A)** and lungs **(B)**. All data were shown as means ± SD, *n* = 3–6. *p < 0.05; ***p <0.001.

In order to assess the initial protective efficacy of LT33 and LT28 vaccines preliminarily, C57BL/6 mice were vaccinated with PBS, BCG, LT33, LT28, LT33+LT28, and LT33+LT28+MH in DPC adjuvant respectively. Subsequently, the mice were challenged with the H37Ra strain *via* tail vein injection 12 weeks after the final vaccination. BCG provided the most effective protection against bacterial growth, reducing the number of viable bacteria in the spleen (CFU) by 1.61 log10 (H37Ra) compared to the PBS group (**Figure 6A**). Neither LT33 nor LT28 alone resulted in a decrease in bacterial load in the spleen or lungs compared to the PBS group (*p* > 0.05) (**Figures 6A, B**). However, co-immunization with LT33 and LT28 led to a reduction of 1.52 log10 CFU in the spleen compared to the PBS group (**Figure 6A**). Co-immunization with LT33, LT28, and MH (12) showed a similar effect in the LT33 and LT28 co-immunized groups (**Figures 6A, B**).

**Figure 7:**
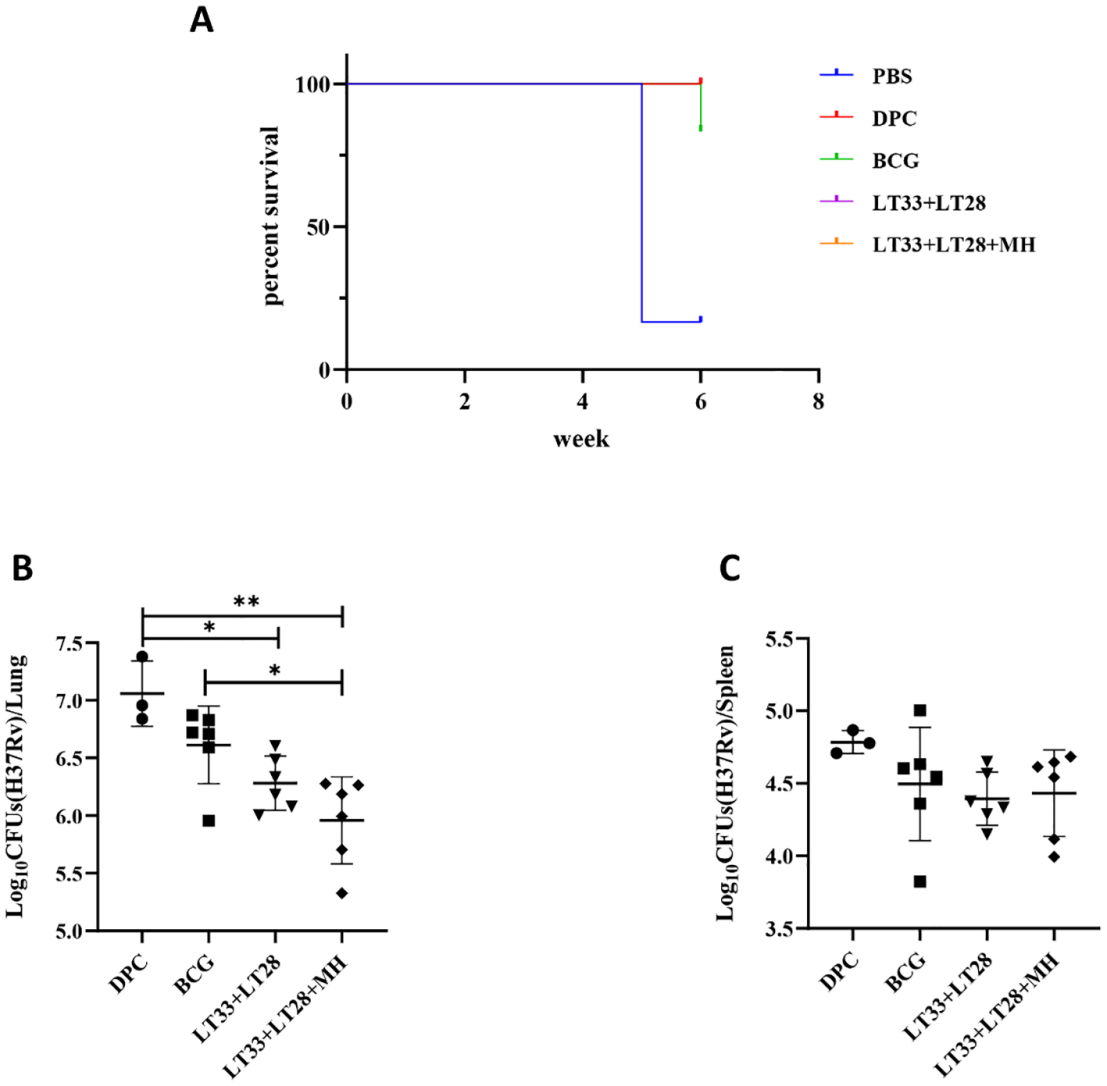
The protective efficacy of LT33 and LT28 challenged with the H37Rv strain. At 12 weeks after the last immunization, the C57BL/6 mice were aerosol-infected with the *M. tuberculosis* H37Rv strain. Survival curves of C57BL/6 mice infected with *M. tuberculosis* H37Rv **(A)**. Mice were euthanized and the bacterial burden (H37Rv) was measured in the lungs **(B)**. Mice were euthanized and the bacterial burden (H37Rv) was measured in the spleen **(C)**. All data were shown as means ± SD, *n* = 3–6. Three mice in DPC groups were used in the quality control for infection. *p < 0.05; **p < 0.01.

To assess the immune protection effect of LT33 and LT28 combination, we evaluated the efficacy of LT33+LT28 and LT33+LT28+MH in protecting against the H37Rv strain by aerosol challenge after 12 weeks post-immunization. Following the H37Rv challenge, vaccinated mice exhibited normal survival rates. In the PBS groups, five out of six mice died at 5 weeks, while one mouse in the BCG group died at 6 weeks (**Figure 7A**). Compared to the DPC control, the LT33+LT28 group demonstrated superior protection against the *M. tuberculosis* H37Rv strain, resulting in a 0.77 log10 CFU reduction in lung bacterial load (**Figure 7B**). Furthermore, the combination of LT33, LT28, and MH provided enhanced protection and caused a 1.09 log10 CFU decrease in lung bacterial load compared to the DPC group (**Figure 7B**). LT33+LT28+MH even outperformed BCG, achieving a 0.65 log10 CFU reduction in the lung bacterial load compared to the BCG group (**Figure 7B**), thereby providing stronger protective efficacy than the combination of MH and the fusion protein BG (29). There were no discernible differences between the vaccine and control groups in terms of spleen response post-H37Rv strain challenge (**Figure 7C**).

**Figure 8:**
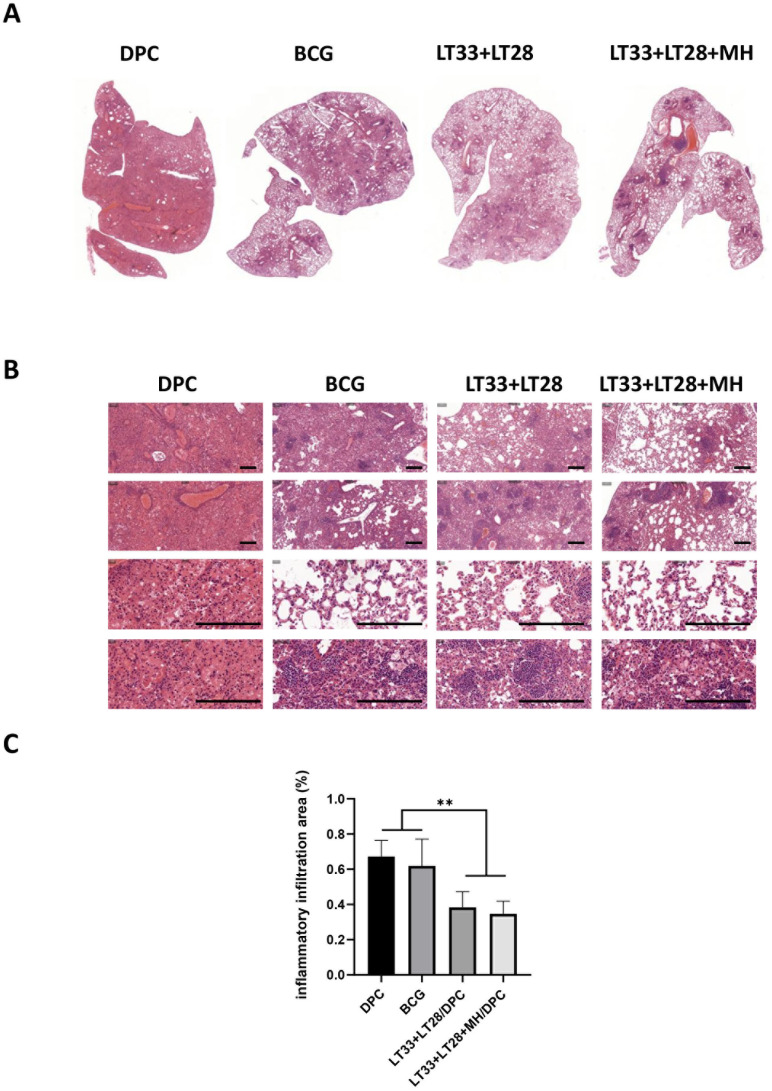
The pathological lesions in vaccine-immunized mice following the H37Rv challenge. **(A)** Global scanning of H&E staining in mouse lungs infected by H37Rv strain. **(B)** Pathological changes in the lungs of mice infected with the H37Rv strain. Scale bars, 200 μm. **(C)** The percentage of the lung area occupied by inflammatory cells. All data were shown as means ± SD, *n* = 3–6. **p < 0.01.

Mice in the DPC group exhibited significant inflammatory exudative lesions in their lungs after *M. tuberculosis* infection (**Figure 8A**). Unlike this, all vaccinated mice showed less pathologic lesions with predominantly lymphoproliferative changes (**Figure 8B**). Mice immunized with the vaccine containing LT33 and LT28 fusion proteins exhibited reduced inflammatory infiltration in the lung compared to the DPC and BCG groups (**Figures 8B, C**).

## 4 Discussion

In this study, we selected specific secreted antigens of *M. tuberculosis* and latency-related antigen to construct two fusion proteins, LT33 and LT28. These fusion proteins were successfully produced and purified in *E. coli* using salting-out and GFC techniques. Immunization with LT33 and LT28 vaccines in mice induced robust cell-mediated and humoral immune responses. The combination of LT33 and LT28 provided effective protection against *M. tuberculosis* infection, compared to control groups receiving PBS or adjuvant alone. In addition, the combination of LT33 and LT28 with another fusion protein, MH, demonstrated superior protective efficacy to BCG vaccination.

The integration of liposomal systems with immunostimulatory agents such as TLR agonists represents a promising strategy for vaccine adjuvant development. We have constructed the DPC adjuvant through which the liposome DDA facilitates the delivery of TB antigens and PolyI:C to antigen-presenting cells (APCs), subsequently modulating immune responses. Mice that received immunization with LT33 or LT28 exhibited strong antigen-specific IFN-γ and IL-2 responses, indicating the induction of a robust Th1-type immune reaction by LT33 and LT28. Importantly, the combination of LT33 and LT28 with the DPC adjuvant promoted the development of long-lived memory T cells. The cell-mediated immune response is considered the primary defense mechanism of the host against *M. tuberculosis* infection (30). It is believed that the Th1-type immune response induced by vaccines triggers phagocytes to engulf intracellular pathogens (31). Following antigen stimulation, T cells can differentiate into T_EFF_, T_EM_, T_CM_, and T_SCM_ cells, but only T_CM_ and T_SCM_ cells can be maintained for a long time (32). After the re-stimulation, T_CM_ can differentiate into T_EM_ and T_eff_ and play an effector role once a pathogen is encountered (33, 34). Long-term immune response depends on the maintenance of memory T cells, which can survive for a long time and has strong proliferative capacity (35). T_CM_- and T_SCM_-mediated long-term immunological memory would be the efficient immune responses induced by vaccination to control *M. tuberculosis* infection (35). The goal of TB vaccine is to establish durable immune protection (36). Our study, along with other research, has indicated that the TB subunit vaccine effectively induced long-lived memory T cells (37). Furthermore, our subunit vaccine candidate, as well as the subunit vaccine Ag85B-ESAT-6/CAF01, induced the generation of IL-2-producing T cells at elevated levels for controlling chronic *M. tuberculosis* infection (38). In addition, the subunit vaccines LT33 and LT28 induced a ratio of antigen-specific antibody subtypes IgG2c to IgG1 that exceeded 1, indicating that these vaccines elicited a Th1-type immune response.

During *M. tuberculosis* infection, the *M. tuberculosis* population exhibits various metabolic states and has the capability to transition between them (39). Some studies indicate that subunit vaccines containing antigens from various metabolic stages offer superior protective efficacy compared to those composed solely of antigens highly expressed during replication (40). Rv1738, upregulated in *M. tuberculosis* latency models under hypoxic conditions, demonstrates strong immunogenicity in latent TB (25, 26). A combination of early protective antigens (ESAT6 and CFP10) with Rv1738, such as in the LT33 vaccine, can effectively target bacteria across different metabolic stages and provide immune defense. The novel subunit vaccine H56/IC31, comprising latency and replicating antigens, demonstrates efficacy in containing infection and preventing reactivation post-exposure (11). Similarly, the multistage subunit vaccine ID93, consisting of the latency-associated antigen Rv1813, exhibits high protective efficacy comparable to BCG.

Utilizing a combination of antigens has the potential to enhance the effectiveness of vaccines against *M. tuberculosis* (41). *M. tuberculosis* antigens can be recognized by a limited set of HLA molecules, and vaccines containing multiple antigens offer the advantage of being identifiable by a diverse range of human populations (13). Expanding the range of antigens can potentially enhance the effectiveness of subunit vaccines (42–44). Immunization of mice with either LT33 or LT28 vaccine alone did not show significant protective effects. However, combining LT33 and LT28 demonstrated improved protection, close to BCG. Furthermore, the addition of MH (45) to the combination of LT33 and LT28 further enhanced vaccine efficacy, resulting in a 0.65 log10 CFU reduction in the lung compared to the BCG group. These findings suggested that the combination of LT33, LT28, and MH would be an ideal antigen combination against *M. tuberculosis* infection.

The bacterial strain, dose, and the route of infection can significantly affect the results of protective efficacy studies. H37Ra, an attenuated strain of *M. tuberculosis*, shares a highly conserved gene content and order with the virulent strain H37Rv. This allows H37Ra to be used for preliminary evaluation of vaccine efficacy with fewer biosafety restrictions (46). However, certain genes associated with pathogen virulence and persistence are either absent or expressed at low levels in H37Ra (47). Mutations in the *phoP* gene contribute to the attenuation of *M. tuberculosis* H37Ra by impeding the secretion of key virulence proteins such as ESAT6 and CFP10 (48). This could explain the lack of protection against H37Ra challenge observed with a single LT33 containing ESAT6 and CFP10, although mice immunized with BCG exhibited reduced bacterial loads following H37Ra challenge compared to controls. In our study, vaccinated mice were challenged with H37Ra *via* the tail vein at a high dose and infected with H37Rv through aerosol at a low dose. Upon intravenous injection of the H37Ra strain, the spleen immune cells in vaccinated mice promptly identified and cleared the H37Ra strain. The H37Rv strain aerosol challenge mimics the natural infection of lung TB, allowing for a more accurate assessment of vaccine response. It is interesting to note that there was no statistically significant decrease in the lung bacterial burden when these BCG-immunized mice were challenged with H37Rv. This disparity in protective efficacy may be attributed to the waned immune responses induced by BCG, as well as the bacterial doses applied in the challenge experiments.

In summary, two tag-free fusion proteins, LT33 and LT28, were successfully engineered and purified. Both proteins demonstrated the ability to elicit promising immune responses individually, while the combined administration of LT33 and LT28 exhibited immune protection against *M. tuberculosis* infection in murine models. Additionally, the inclusion of another fusion protein, MH, further enhanced the protective efficacy. These findings suggest that the incorporation of LT33 and LT28, with or without MH, into a DPC formulation could represent a promising candidate for TB vaccine.

## Data Availability

The original contributions presented in the study are included in the article/**Supplementary Material**. Further inquiries can be directed to the corresponding authors.

## Supplementary material

Supplementary Figure S1 Flow cytometry gating strategy. (A) In the intracellular cytokine staining assay, spleen lymphocytes were labeled with anti-CD4-FITC, anti-CD8-PerCP-Cy5.5, anti-IFN-γ-APC, and anti-IL-2-PE antibodies. Initially, the lymphocytes were gated by SSC-H and FSC-H parameters, followed by the selection of single cells through FSC-H and FSC-A. Ultimately, the analysis via flow cytometry focused on CD4^+^ IFN-γ^+^ T cells, CD4^+^ IL-2^+^ T cells, CD8^+^ IFN-γ^+^ T cells and CD8^+^ IL-2^+^ T cells. (B) The raw flow plot of PBS abnormal group stimulated with ESAT6.

Supplementary Figure S2 The MFI of IFN-γ. The MFI of IFN-γ were analyzed by the software of NovoExpress. (A) The MFI of IFN-γ were analyzed from **Figure 3A**. (B) The MFI of IFN-γ were analyzed from **Figure 3B**. (C) The MFI of IFN-γ were analyzed from **Figure 4**. All data were shown as means ± SD, n=3-4. *p < 0.05; ** p < 0.01.

Supplementary Figure S3 Flow cytometric analysis of IFN-γ and IL-2 producing T cells from LT33 immunized mice. At 6 weeks after the last immunization, the splenic lymphocytes were separated and stimulated with mixed antigens of ESAT6, CFP10 and Rv1738 *in vitro* for 12 h. The intracellular cytokines staining was analyzed using flow cytometry.

Supplementary Figure S4 Flow cytometric analysis of IL-2 producing T cells from LT28 immunized mice. At 12 weeks after the last immunization, the splenic lymphocytes were separated and stimulated with single antigen (MPT64, Rv1978 and Rv2645) *in vitro* for 12 h. Subsequently, the intracellular cytokines staining was analyzed using flow cytometry. (A) Flow cytometric analysis of IL-2 producing CD4^+^ T cells. (B) Statistical analysis of the proportion of IL-2 producing CD4^+^ T cells. Results are presented as means ± SD, *n* = 4. ****p* < 0.005.

Supplementary Figure S5 Cytometric plot for the purity check.

Supplementary Figure S6 The raw flow plots of the control and vaccine groups stimulated with MPT64, Rv1978, or Rv2645.

Supplementary Figure S7 Expression of LT28 verified with polyacrylamide gel electrophoresis. E. coli BL21 expressing LT28 lysate (lane 1), Centrifuging supernatant liquid from LT28 lysate (lane 2), Centrifugal precipitation of LT28 lysate liquid (lane 2), E. coli BL21 lysate (lane 4).
