# Supplementary figures and images for: The combination of Mycobacterium tuberculosis fusion proteins LT33 and LT28 induced strong protective immunity in mice
Source: Front Immunol. 2024 Nov 22;15:1450124. doi: 10.3389/fimmu.2024.1450124 (PMC11621036; doi:10.3389/fimmu.2024.1450124)

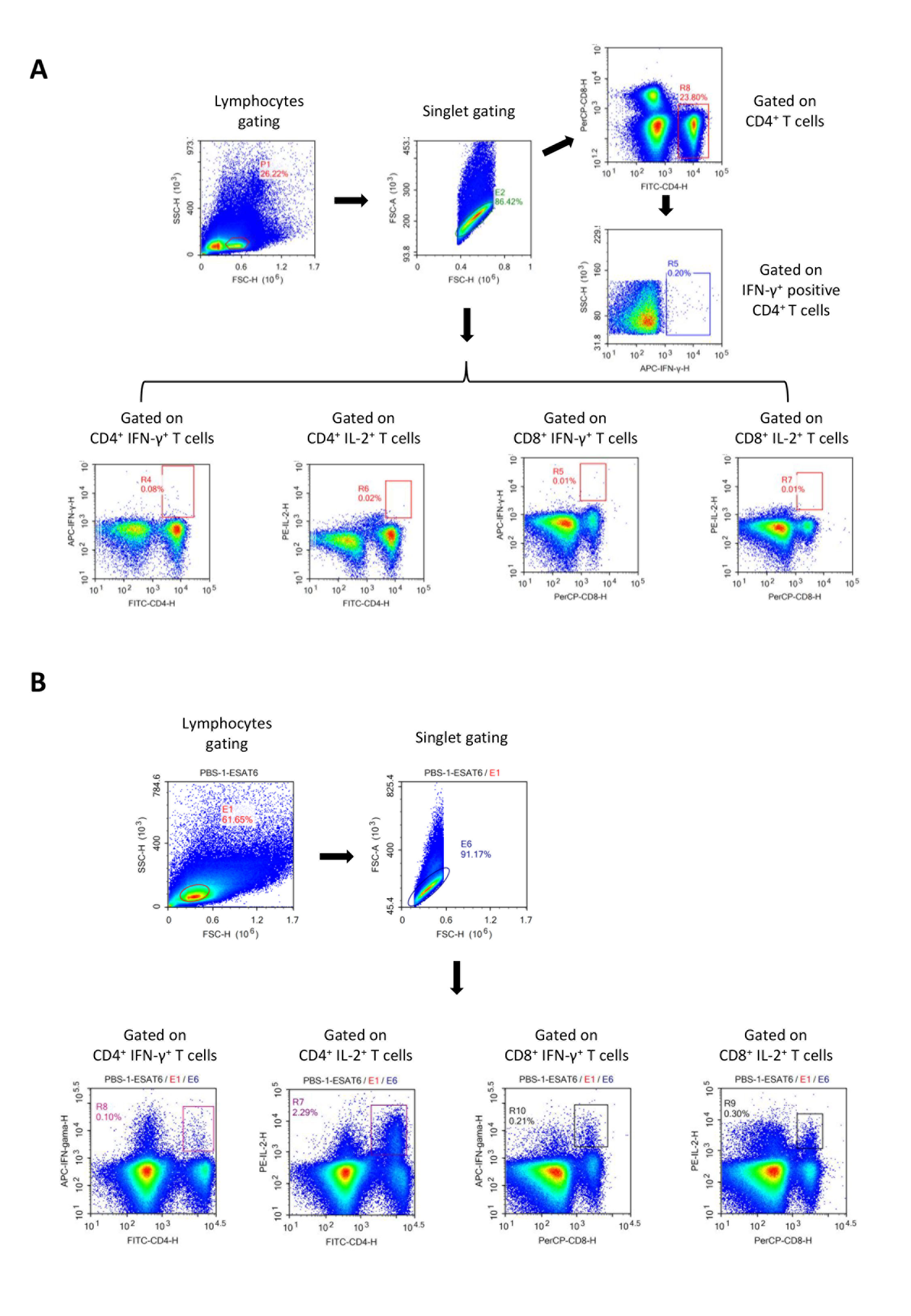

Supplement: Supplementary Figure S1 — Flow cytometry gating strategy. (A) In the intracellular cytokine staining assay, spleen lymphocytes were labeled with anti-CD4-FITC, anti-CD8-PerCP-Cy5.5, anti-IFN-γ-APC, and anti-IL-2-PE antibodies. Initially, the lymphocytes were gated by SSC-H and FSC-H parameters, followed by the selection of single cells through FSC-H and FSC-A. Ultimately, the analysis via flow cytometry focused on CD4+ IFN-γ+ T cells, CD4+ IL-2+ T cells, CD8+ IFN-γ+ T cells and CD8+ IL-2+ T cells. (B) The raw flow plot of PBS abnormal group stimulated with ESAT6. [file Image1.tif]

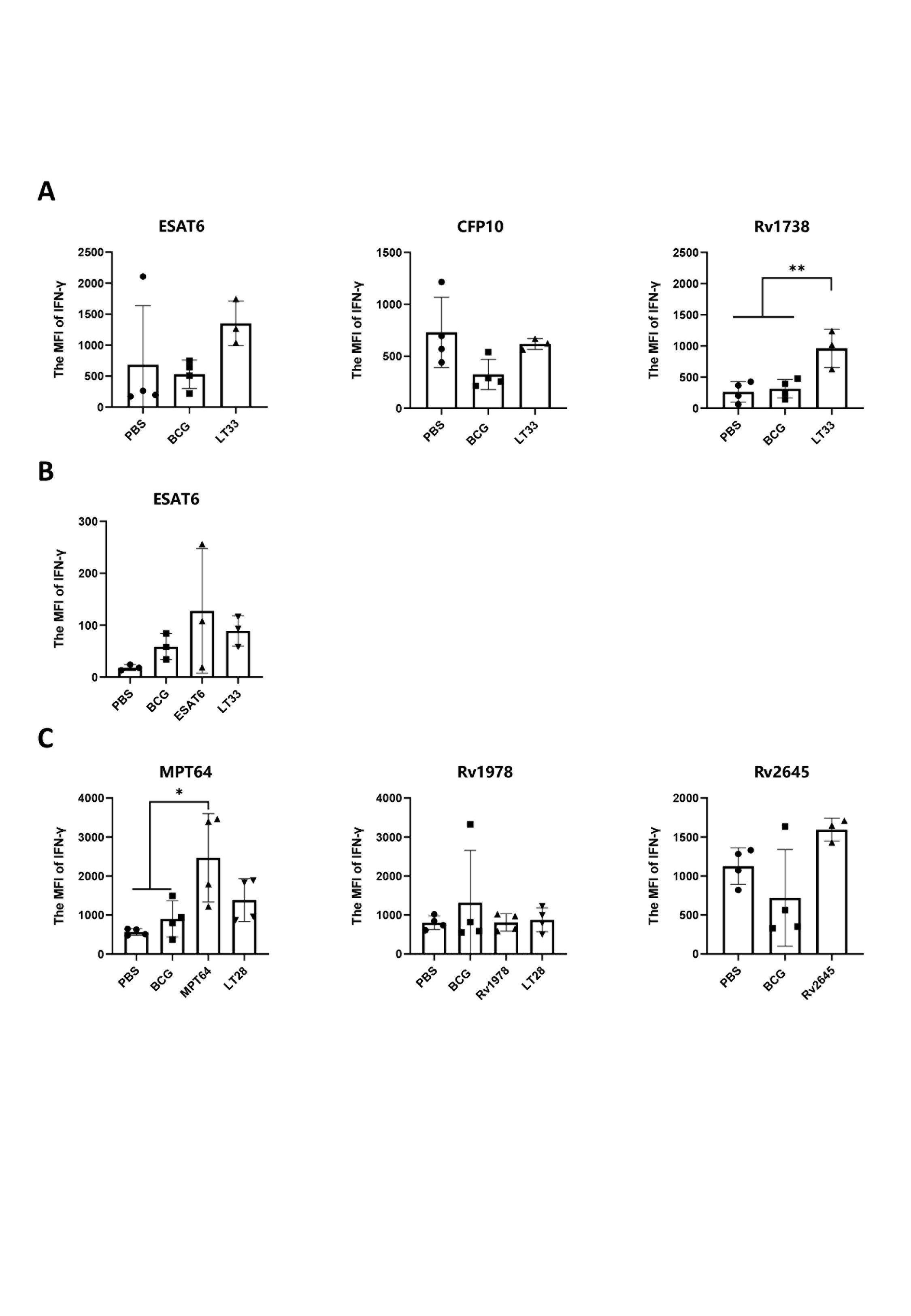

Supplement: Supplementary Figure S2 — The MFI of IFN-γ. The MFI of IFN-γ were analyzed by the software of NovoExpress. (A) The MFI of IFN-γ were analyzed from Figure 3A . (B) The MFI of IFN-γ were analyzed from Figure 3B . (C) The MFI of IFN-γ were analyzed from Figure 4 . All data were shown as means ± SD, n=3-4. *p < 0.05; ** p < 0.01. [file Image2.tif]

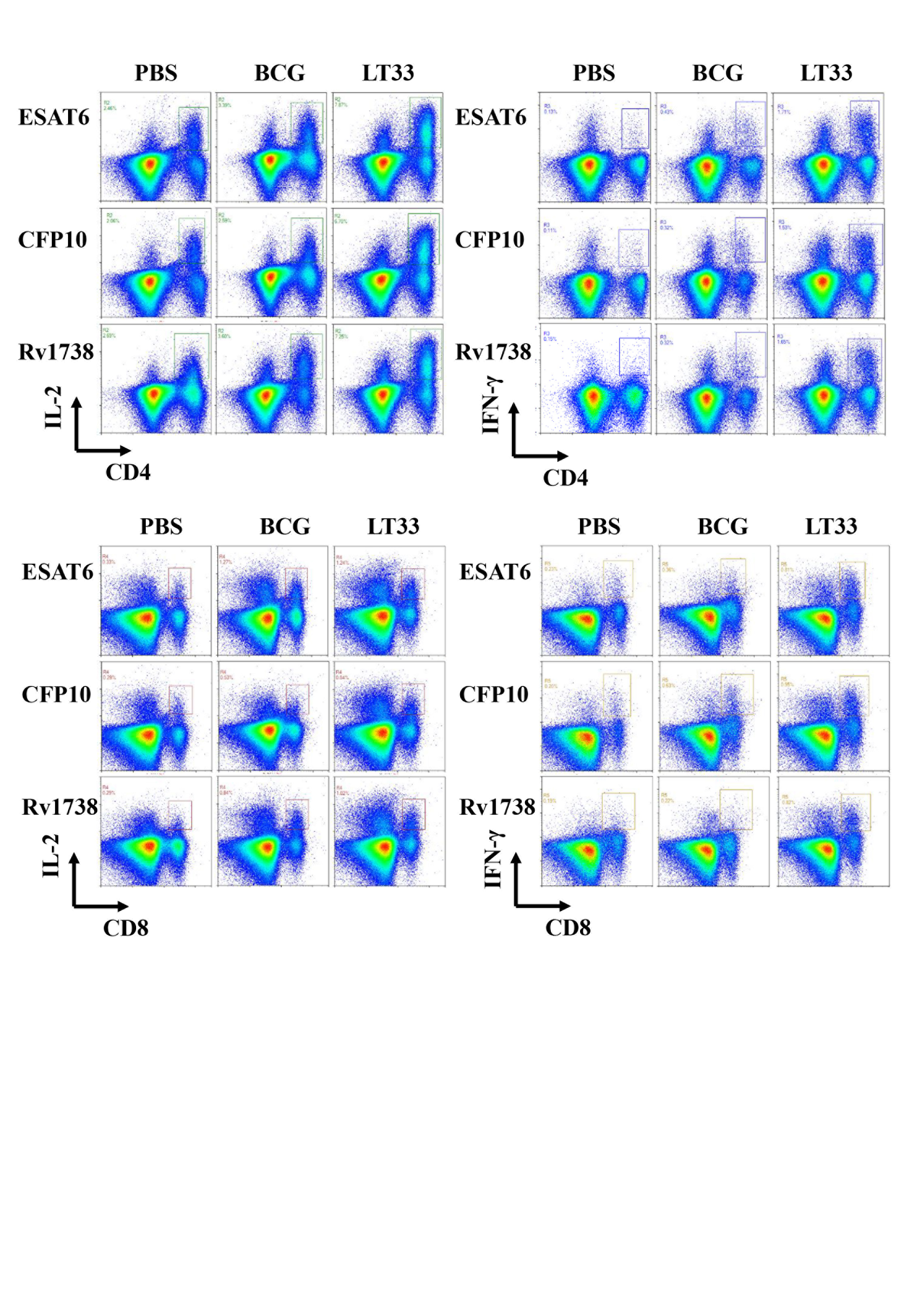

Supplement: Supplementary Figure S3 — Flow cytometric analysis of IFN-γ and IL-2 producing T cells from LT33 immunized mice. At 6 weeks after the last immunization, the splenic lymphocytes were separated and stimulated with mixed antigens of ESAT6, CFP10 and Rv1738 in vitro for 12 h. The intracellular cytokines staining was analyzed using flow cytometry. [file Image3.tif]

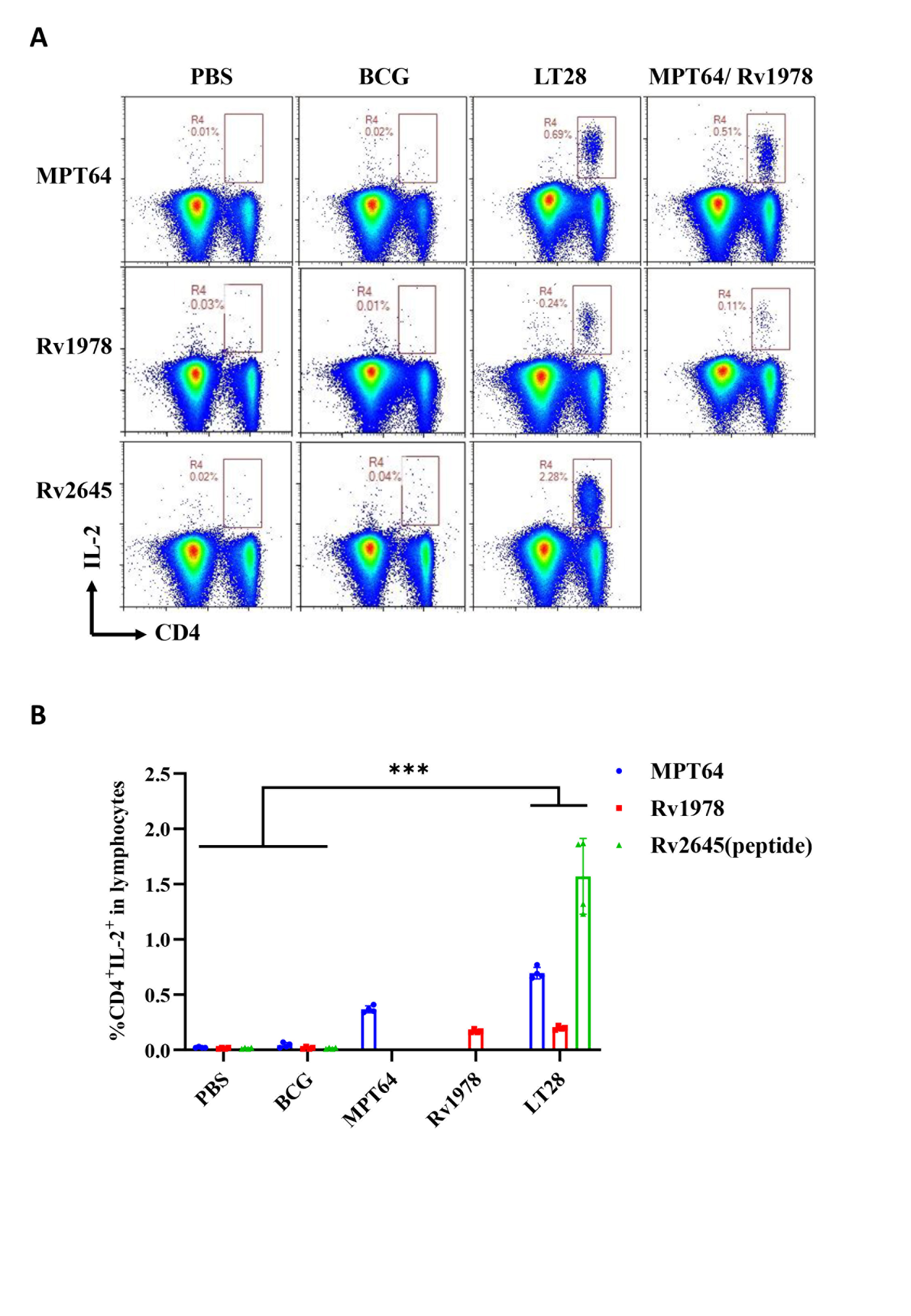

Supplement: Supplementary Figure S4 — Flow cytometric analysis of IL-2 producing T cells from LT28 immunized mice. At 12 weeks after the last immunization, the splenic lymphocytes were separated and stimulated with single antigen (MPT64, Rv1978 and Rv2645) in vitro for 12 h. Subsequently, the intracellular cytokines staining was analyzed using flow cytometry. (A) Flow cytometric analysis of IL-2 producing CD4+ T cells. (B) Statistical analysis of the proportion of IL-2 producing CD4+ T cells. Results are presented as means ± SD, n = 4. ***p < 0.005. [file Image4.tif]

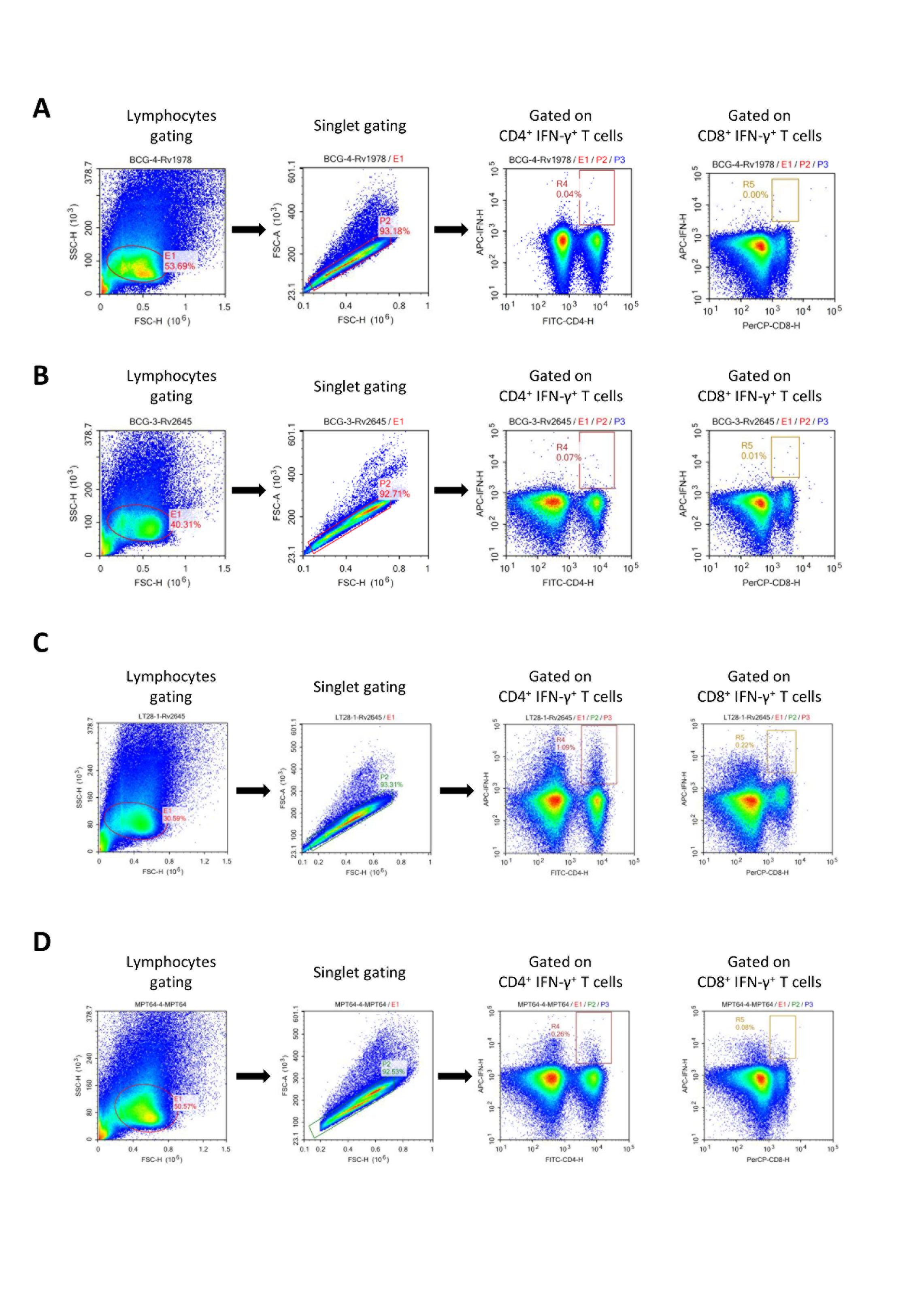

Supplement: Supplementary Figure S5 — Cytometric plot for the purity check. [file Image5.tif]

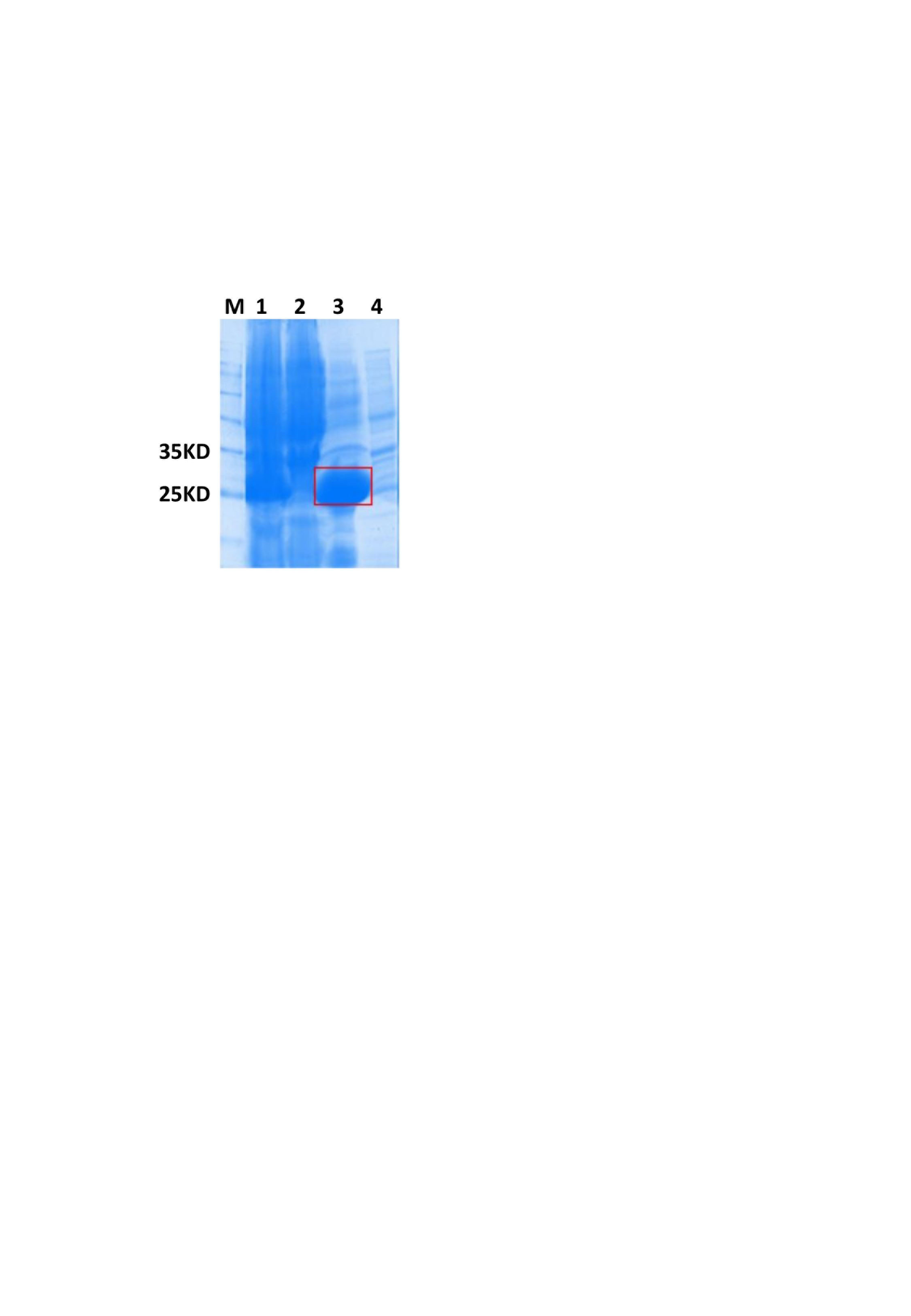

Supplement: Supplementary Figure S6 — The raw flow plots of the control and vaccine groups stimulated with MPT64, Rv1978, or Rv2645. [file Image6.tif]

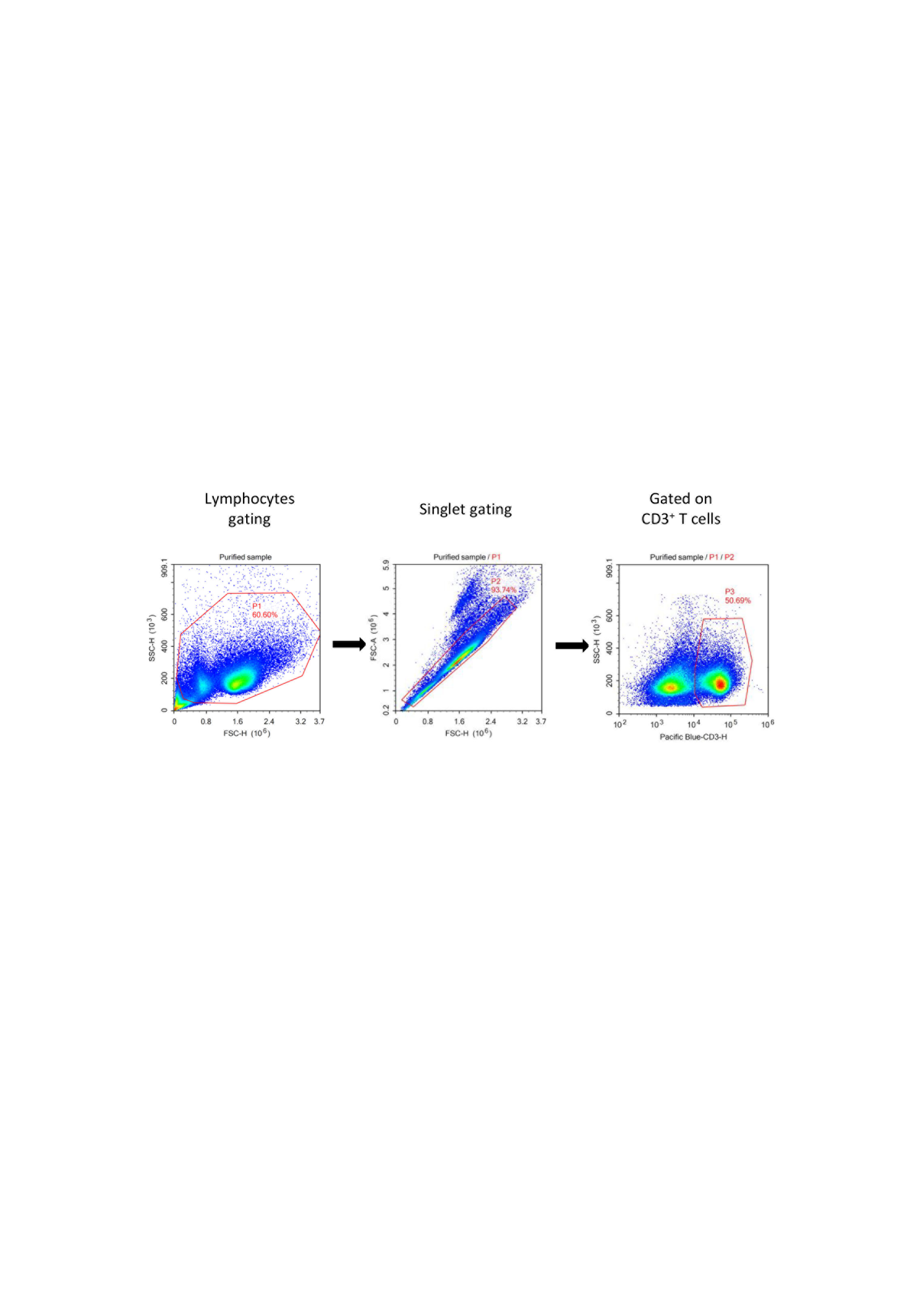

Supplement: Supplementary Figure S7 — Expression of LT28 verified with polyacrylamide gel electrophoresis. E. coli BL21 expressing LT28 lysate (lane 1), Centrifuging supernatant liquid from LT28 lysate (lane 2), Centrifugal precipitation of LT28 lysate liquid (lane 2), E. coli BL21 lysate (lane 4). [file Image7.tif]
