# Supplementary material for: The combination of Mycobacterium tuberculosis fusion proteins LT33 and LT28 induced strong protective immunity in mice
Source: Front Immunol. 2024 Nov 22;15:1450124. doi: 10.3389/fimmu.2024.1450124 (PMC11621036; doi:10.3389/fimmu.2024.1450124)
Supplement: Supplementary file 9 [file DataSheet1.docx]

Supplementary Material

# Supplementary Data

# Supplementary Figures and Tables

## Supplementary Figures


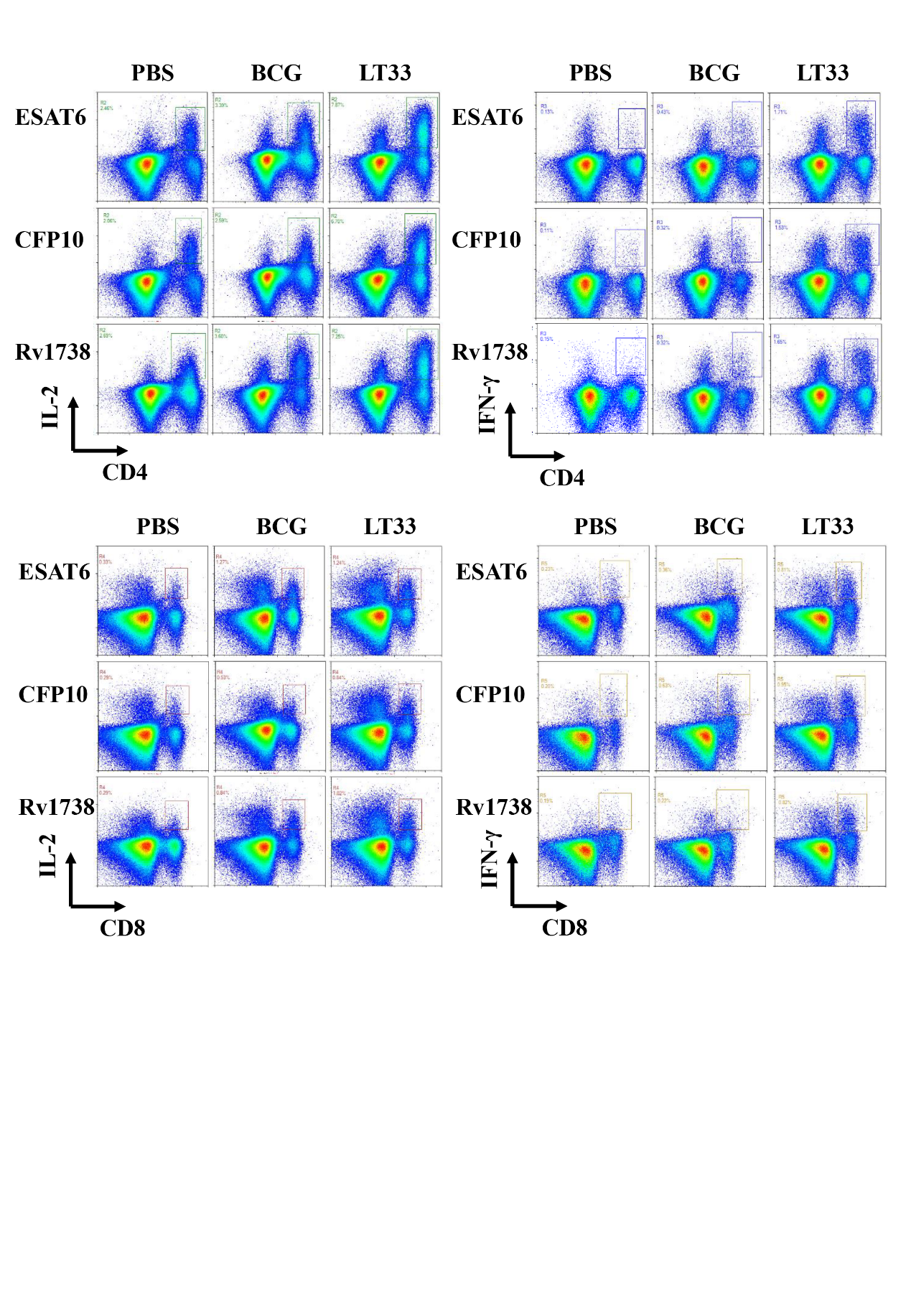


Figure S1. Flow cytometric analysis of IFN-γ and IL-2 producing T cells from LT33 immunized mice.

At 6 weeks after the last immunization, the splenic lymphocytes were separated and stimulated with mixed antigens of ESAT6, CFP10 and Rv1738 in vitro for 12 hours. The intracellular cytokines staining was analyzed using flow cytometry.


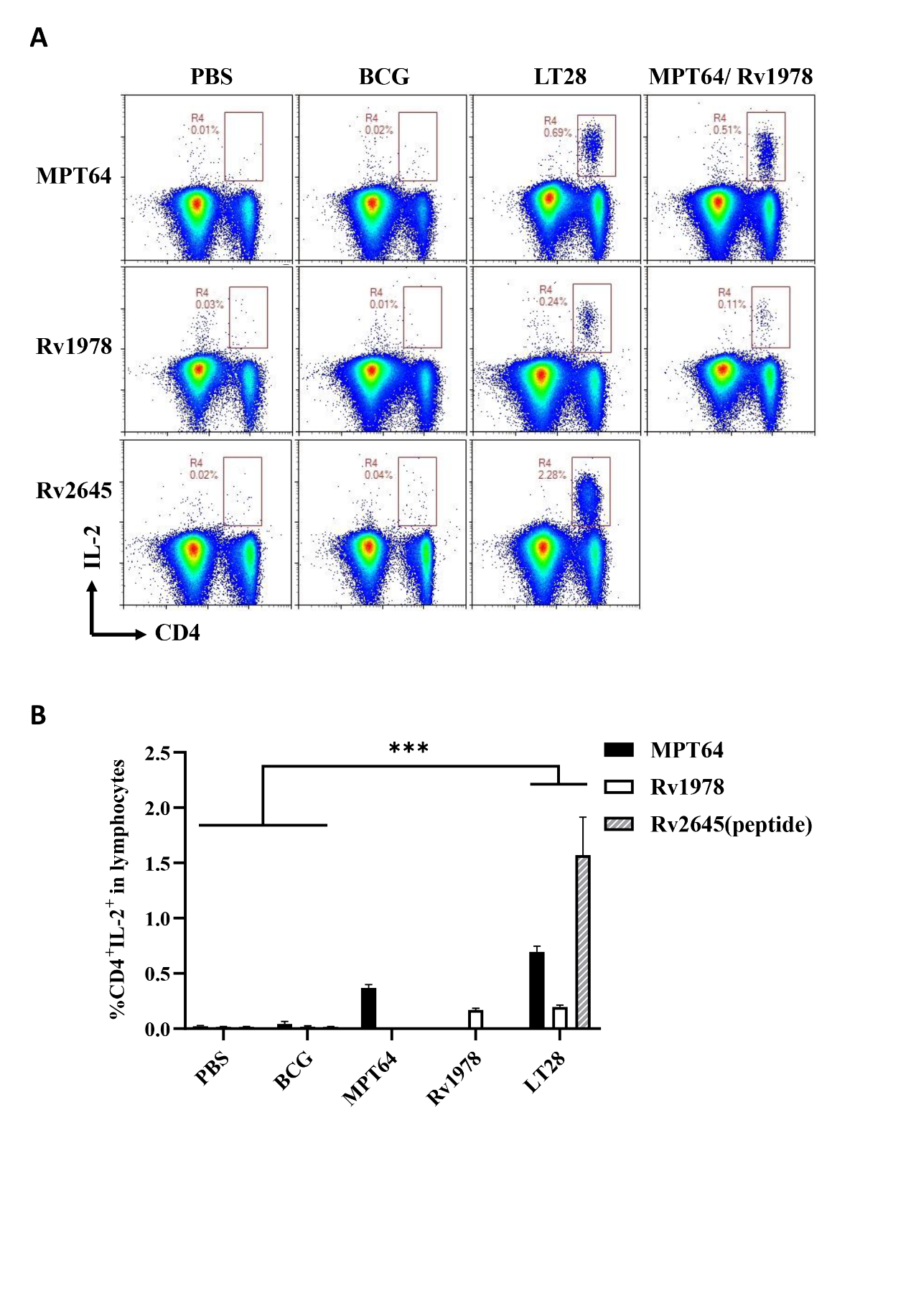


Figure S2. Flow cytometric analysis of IL-2 producing T cells from LT28 immunized mice.

At 12 weeks after the last immunization, the splenic lymphocytes were separated and stimulated with single antigen (MPT64, Rv1978 and Rv2645) in vitro for 12 hours. The intracellular cytokines staining was analyzed using flow cytometry. (A) Flow cytometric analysis of IL-2 producing CD4^+^ T cells. (B) Statistical analysis of the proportion of IL-2 producing CD4^+^ T cells. Results are presented as means ± SD, *n* = 4. *** *p* < 0.005.
