# Supplementary material for: The combination of Mycobacterium tuberculosis fusion proteins LT33 and LT28 induced strong protective immunity in mice
Source: Front Immunol. 2024 Nov 22;15:1450124. doi: 10.3389/fimmu.2024.1450124 (PMC11621036; doi:10.3389/fimmu.2024.1450124)
Supplement: Supplementary file 11 [file DataSheet2.docx]

Statement of resubmission

We have submitted a manuscript entitled “Construction of Mycobacterium tuberculosis fusion proteins LT33 and LT28 and their immune protective efficacy in mice” to Frontiers in Immunology as a research paper On April 2, 2024. At May 3rd, 2024, this manuscript was rejected. This manuscript was rejected because it didn't upload complete original data in the last manuscript submitted. We supplemented the original data and resubmitted it this time.
