# Supplementary material for: The combination of Mycobacterium tuberculosis fusion proteins LT33 and LT28 induced strong protective immunity in mice
Source: Front Immunol. 2024 Nov 22;15:1450124. doi: 10.3389/fimmu.2024.1450124 (PMC11621036; doi:10.3389/fimmu.2024.1450124)
Supplement: Supplementary file 13 [file DataSheet3.docx]

**Expression of LT33 in *E. coli*.**


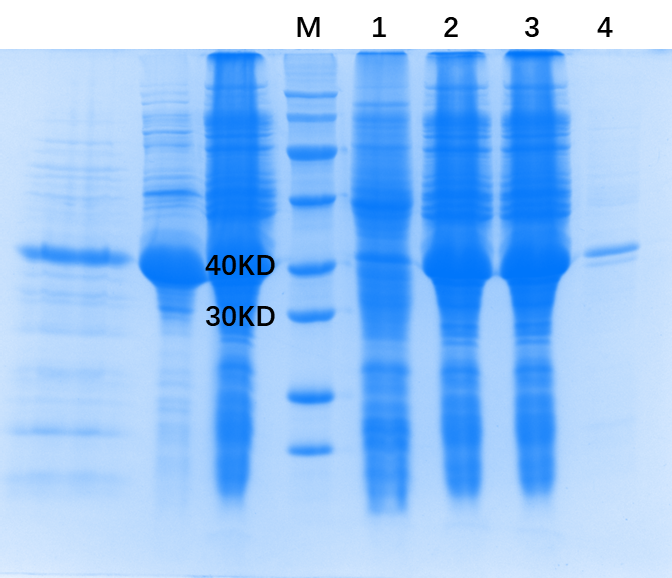


Total *E. coli* lysate of BL21 strain (lane 1), total *E. coli* lysate (lane 2), supernatant of *E. coli* lysate (lane 3), and sediment of E. coli lysate (lane 4), M, molecular weight.

**Purified fusion proteins LT33**


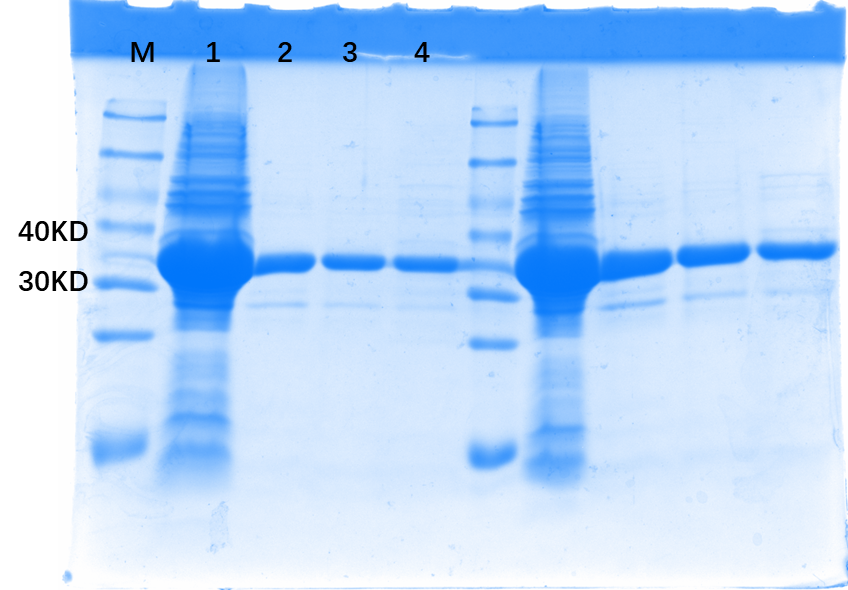


Purification of LT33 verified with polyacrylamide gel electrophoresis. *E. coli* BL21 expressing LT33 lysate (lane 1), Purification of LT33 (lane 2,3,4), M, molecular weight.

**Expression of LT28 in *E. coli*.**


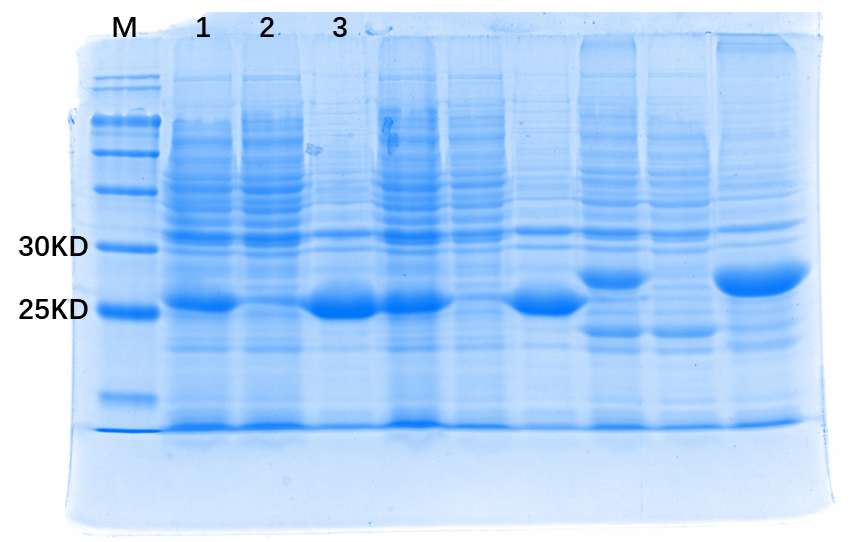


Total *E. coli* lysate (lane 1), supernatant of *E. coli* lysate (lane 2), and sediment of E. coli lysate (lane 3), M, molecular weight.

**Purified fusion proteins LT28**


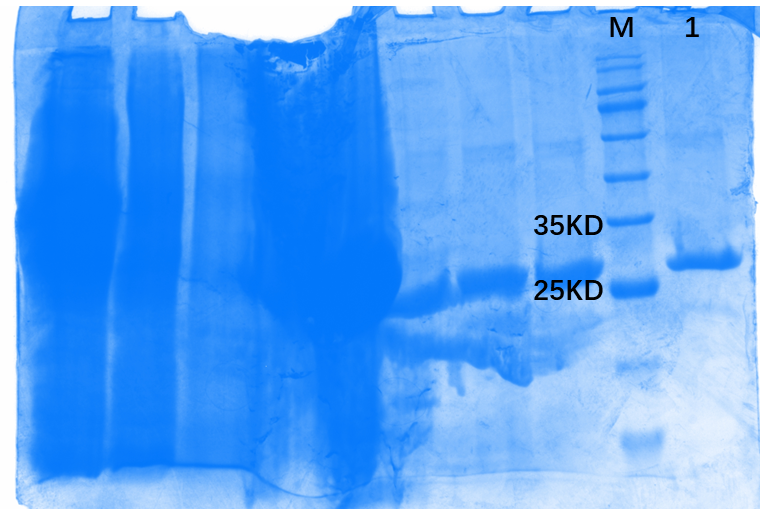


Purification of LT28 verified with polyacrylamide gel electrophoresis. Purification of LT28 (lane 1), M, molecular weight.

**Purified LT33 was verified by immunoblot.**

**
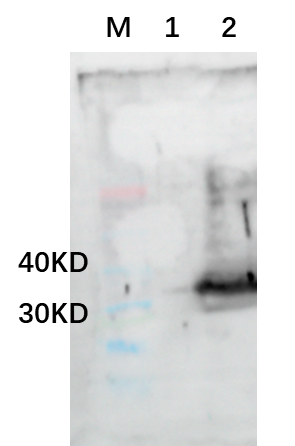
**

Purified LT33 was verified by immunoblot. Negative control (lane 1), Mouse polyclonal anti-ESAT6 (lane 2), M, molecular weight.

**Purified LT28 was verified by immunoblot.**


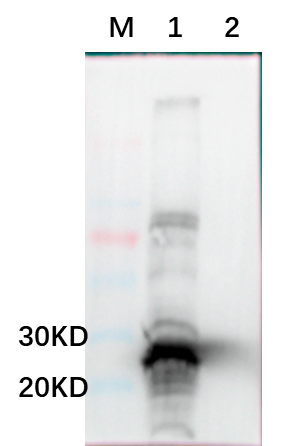


Purified LT28 was verified by immunoblot with anti-MPT64 serum. Mouse polyclonal anti-MPT64 (lane 1), Negative control (lane 2), M, molecular weight.
